# Slow Co-Evolution of the MAGO and Y14 Protein Families Is Required for the Maintenance of Their Obligate Heterodimerization Mode

**DOI:** 10.1371/journal.pone.0084842

**Published:** 2014-01-08

**Authors:** Pichang Gong, Man Zhao, Chaoying He

**Affiliations:** 1 State Key Laboratory of Systematic and Evolutionary Botany, Institute of Botany, Chinese Academy of Sciences, Beijing, China; 2 University of Chinese Academy of Sciences, Beijing, China; Oxford Brookes University, United Kingdom

## Abstract

The exon junction complex (EJC) plays important roles in RNA metabolisms and the development of eukaryotic organisms. MAGO (short form of MAGO NASHI) and Y14 (also Tsunagi or RBM8) are the EJC core components. Their biological roles have been well investigated in various species, but the evolutionary patterns of the two gene families and their protein-protein interactions are poorly known. Genome-wide survey suggested that the *MAGO* and *Y14* two gene families originated in eukaryotic organisms with the maintenance of a low copy. We found that the two protein families evolved slowly; however, the MAGO family under stringent purifying selection evolved more slowly than the Y14 family that was under relative relaxed purifying selection. MAGO and Y14 were obliged to form heterodimer in a eukaryotic organism, and this obligate mode was plesiomorphic. Lack of binding of MAGO to Y14 as functional barrier was observed only among distantly species, suggesting that a slow co-evolution of the two protein families. Inter-protein co-evolutionary signal was further quantified in analyses of the *Tol-MirroTree* and co-evolution analysis using protein sequences. About 20% of the 41 significantly correlated mutation groups (involving 97 residues) predicted between the two families was clade-specific. Moreover, around half of the predicted co-evolved groups and nearly all clade-specific residues fell into the minimal interaction domains of the two protein families. The mutagenesis effects of the clade-specific residues strengthened that the co-evolution is required for obligate MAGO-Y14 heterodimerization mode. In turn, the obliged heterodimerization in an organism serves as a strong functional constraint for the co-evolution of the MAGO and Y14 families. Such a co-evolution allows maintaining the interaction between the proteins through large evolutionary time scales. Our work shed a light on functional evolution of the EJC genes in eukaryotes, and facilitates to understand the co-evolutionary processes among protein families.

## Introduction

The exon junction complex (EJC) is involved in mRNA intracellular export, cytoplasmic localization, non-sense mediated mRNA decay, and translation enhancement in metazoan and plants [1]–[6]. The complex includes more than 10 different proteins, such as MAGO (short form of MAGO NASHI), Y14 (also Tsunagi or RBM8), eIF4A-III and BTZ (short form of Barentsz, also MLN51) [7]–[12]. MAGO and Y14 are core components of the EJC [1], [9], [13]. Both are nuclear-cytoplasm shuttling proteins, and Y14 has a central RNA binding domain [8], [14], [15]. The accumulating evidence demonstrated that the genes encoding these proteins have acquired essential roles in the development of animals and plants. In *Drosophila*, a single point mutant in the *MAGO* locus gives rise to a *grandchildless* phenotype due to a defect in the correct cytoplasmic localization of *oskar* mRNA [16]–[18]. The mutation leads to several developmental defects including improper development of the posterior lobe of the embryo, non-viable egg sacs in female offspring, impairment of germ plasm cell polarity, and germline stem cell differentiation [16], [18], [19]. In *Caenorhabditis elegans*, knock down of *mag-1* causes masculinization of the germ line in RNA-injected hermaphrodites, suggesting that this gene is involved in hermaphrodite germ-line sex determination [20]. In mouse, downregulating *MAGO* causes a cold-sensitive defect in cell cycle transition indicating that *MAGO* gene is related to cell cycle regulation [21]. In plants, the orthologs of *MAGO* genes are linked with male fertility. PFMAGO proteins interact with MADS-domain protein MPF2 and are responsible for male fertility in *Physalis* [22]. In *Arabidopsis AtMago* gene is required for pollen grain development and its knockout is lethal [12], [23]. Mutation of *Mv-mago* disrupts spermatogenesis in the water fern *Marsilea vestita* [5], [24]. *MAGO* genes are also involved in other organ development. *AtMago* is required for development of root, shoot, floral meristem and seed in *Arabidopsis* [12]. Overexpression of *TcMago* that is preferentially expressed in root hairs in *Taiwania cryptomerioides* produced taller transgenic tobacco plants with increased root hairs [25]. *Tsunagi*, the *Drosophila* Y14 ortholog [14], is essential for proper localization of *oskar* mRNA [10], [26]. It has roles in embryogenesis and germline sexual switching in *C.elegans* [27] and in regulating oocyte specification in *Drosophila* [19]. In plants, *Y14* genes seem to share common and broad expression domains with *MAGO* genes [6], [12], [25]. Knockdowns of *AtY14* yields a lethal phenotype in *Arabidopsis* [12] suggesting the necessity of the gene in plant development.

These work revealed that MAGO and Y14 have shared functions among animals and plants in embryogenesis and gametophyte development. However, the evolutions of these two protein families are not investigated yet in eukaryotic organisms. Nonetheless, MAGO and Y14 proteins, as the EJC core components, form the heterodimers in all investigated organisms [1], [7], [9], [15], [22], [24], [25], [27]–[29]. The heterodimerization is required for the proper roles [19], [30], [31], [32], and hence these proteins seem to be obligated to form heterodimer throughout evolution of the organisms. To maintain the functional interactions throughout evolution, two different strategies were evolved. Conservation in sequences of both protein families works for the maintenance; alternatively, the coordinated sequence changes in both proteins are required. The later evolutionary process is also coined as co-evolution, a concept borrowed from the interactions between living organisms, like pollinators and flowering plants [33], [34]. Molecular co-evolution including intra- and inter-proteins has become as one of the hotspots in studying the evolution of gene families [35]–[38] since molecular interactions are the underlying basis for biological processes. However, the functional proofs and importance for the co-evolution of the protein families are limited [39]–[41].

How do the MAGO and Y14 protein families and their interactions evolve across eukaryotes? In this work, we hypothesize that the MAGO and Y14 protein families co-evolve in order to maintain the interaction through large evolutionary time scales, and we thoroughly tested this hypothesis by performing computational as well as experimental studies. We observed that the MAGO and Y14 protein families were under different purifying selection forces with unequal evolutionary rates, but they had co-evolved. The experimental evidence confirmed that that co-evolution of the two protein families plays a vital role in the maintenance of the heterodimerization mode. Thus, the provided data reveals novel aspects on the evolutions of MAGO-Y14 system. The origin, functional implications and particularity of the co-evolution of the MAGO and Y14 protein families were discussed. Our data could contribute to understand the functional evolution of the EJC.

## Materials and Methods

### Yeast Two-Hybrid Assays

Leaves of the rice *O. sativa* L. cv. ‘Zhonghua 10’, *Arabidopsis thaliana* (Col), *Solanum lycopersicum*, *Physalis floridana* and *P.philadelphica* were harvested for total RNA isolation. The ORFs of the *MAGO*, *Y14* and *LFY* genes were amplified through routine RT-PCRs. The deleted versions and the site-directed mutations of the *MAGO* and *Y14* genes were generated from the sequenced plasmid. The sequence information of the gene-specific primers is available in Table S1 and Table S2 in File S1. The PCR products were cloned into the pGADT7 or PGBKT7 vector. The non-lethal β-galactosidase activity was performed on the SD/-Trp-Leu-His-Ade plates in the yeast strain AH109. The same amounts of the transformed yeast cells selected on the SD/-Trp-Leu plates were used in the SD/-Trp-Leu-His-Ade conditions for all combinations. The strength of the indicated protein-protein interactions was quantified using the o-nitrophenyl-β-D-galactoside (ONPG) as substrate. The procedures described in Yeast Protocols Handbook (Clontech, Mountain View, USA) were followed.

### Phylogenetic Reconstructions

The multiple sequence alignment (MSA) was performed by Clustal X (http://www.ebi.ac.uk/clustalw) with parameters of Weight matrix: Gonet; Gap opening penalty: 10.0 and Gap extension penalty: 0.20. The phylogenetic trees were constructed by MEGA 5.0 software (http://megasotetware.met/index.html) [42] and PhyML v3.0 [43] using Maximum Likelihood (ML) method with parameters of kimura2-parameter model, partial deletion, and bootstrap (1000 replicates; random seed), Neighbor-Joining method with parameters of Maximum Composite Likelihood model, pairwise deletion, and bootstrap (1000 replicates; random seed) and Maximum Parsimony method with parameters of Close-Neighbor-Interchange (CNI) on Random Trees model partial deletion, and bootstrap (1000 replicates; random seed). These trees had similar topologies, and the ML trees of the MAGO, Y14 and LFY families were presented. The MSAs of these protein families are presented in Dataset S1, S2 and S3, respectively. We used the 18s rRNA to correct the speciation influences, and some other controls such as the non-interaction pairs of the Y14 and MAGO families with the LYF family.

### Evaluation of Genetic Distance and Correlation Analyses

Genetic distances were generated from the multiple alignments using MEGA5 [42]. In order to quantify the co-evolution of interaction proteins, we employed a linear regression analysis measuring the correlation between pairwise evolutionary distances among all proteins in a multiple sequence alignment [44]. Two two-dimensional matrixes X and Y were constructed for the Y14 and MAGO families (X and Y were constructed as N×N matrix). Xij is the pairwise distance between sequence mi and sequence mj, Yij signifies the sequence ni and nj (where ni is bind to mi, and nj is bind to mj). In order to represent multiple Y14s that bind to a single MAGO, *visa versa*, MAGO or Y14 was represented more than once in the matrix in some instances. We used Mantel test [45] to compute the Pearson correlation coefficient *R*.

### Evolutionary Rate and Selection Evaluation

The *dN* and *dS* was respectively calculated with the Kumar method (Kimura 2-para) model [46], [47]. The evolutionary rates ω (*dN/dS*) of the MAGO and Y14 families were calculated in MEGA 5 [42]. The selective pressures acting on these genes were determined using the maximum likelihood method implemented in the CODEML program of the PAML 4 package [45], [48]. The ancestor sequences of the Y14 and MAGO in cereals were reconstructed by Codeml program [45].

### Structural Modeling

The tertiary structures and the interaction interface of OsMAGO1, OsMAGO2, OsY14a and OsY14b and their minimal interaction domains were predicted using the SWISS-MODEL Protein Modeling Server (http://swissmodel.expasy.ory//SWISS-MODEL.html). The structural quality was checked using the programs provided by the same server (Anolea/Gromos), and further supported by the global model quality estimation scores QMEAN4 [49]. The interaction prediction was viewed and edited by the DeepView-SwissPDB-Viewer program.

### Ancestral Character-State Reconstruction

To investigate the diversification of the interaction patterns between the Y14 and MAGO proteins across the species, we conducted character-state reconstructions in Mesquite version 2.75 (http://mesquiteproject.org). In the analysis, every species with available interaction data were included, and topologies indicating the phylogenetic relationships of these species were used as input trees. For each species, one of the two states (0 for absence of interaction and 1 for presence of interaction) were assumed and mapped onto the phylogenetic trees if there is only one gene or if two or more paralogous genes are involved in the interaction. Ancestral states of the protein-protein interaction at the ancestral nodes of each phylogenetic tree were traced by using both likelihood and parsimony methods in the “Trace Character History” function of Mesquite.

### Detecting the Co-Evolved Amino Acids and Their Groups

Co-evolution analysis using protein sequences (CAPS) compares the correlated variance of the evolutionary rates at two sites in multiple sequence alignment (MSA) by comparing the transition probabilities between each pair of amino acids at the two sites, using the BLOSUM substitution matrix [50]. Because sequences that diverged longer ago are more likely to fix mutations at two sites by chance than those diverged recently, BLOSUM values were normalized by the time of divergence between sequences using Poisson corrected amino acid distances. The co-evolution between two sites was then estimated as the correlation in the pairwise amino acid variability, relative to the mean variability per site. Correlated mutation pairs were grouped based on their connectivity to each other; only those “correlated groups” were analyzed. The protein sequences of the Y14 and MAGO families were aligned using Clustal X. Co-evolution analyses were performed using the program CAPSv1.0 [51]. The clade-specific residues were distracted from the large MSAs (Dataset S1 and S2).

### Sequencing and Bioinformatics Analyses

Most sequences of the *MAGO*, *Y14*, *LFY* and *18s rRNA* families were downloaded from the Databases of the NCBI (http://www.ncbi.nlm.nih.gov/BLAST/) and the Phytozome (http://www.phytozome.net/search.php). The information of sequences analyzed is available in Table S3 in File S1. The protein logos were generated using the WebLogo software (http://weblogo.berkeley.edu/logo.cgi). The cDNAs that were experimentally used in the present work were cloned into the pGEM-T EASY vector (Promega, USA) and sequenced. The sequences isolated in this work were deposited into the NCBI database under accession numbers of KF051000-KF051011 (*MAGO* genes), KF051012-KF051021 (*Y14* genes) and KF051022-KF051023 (*LFY* genes).

## Results

### Genome-Wide Survey of the *MAGO* and *Y14* Genes

A comprehensive survey of all the available databases strongly suggested that the *MAGO* and *Y14* two gene families are specific in eukaryotes. The sequences from 36 representative eukaryotic organisms were included in our further analyses (Table S3 in File S1). Gene copy number for each gene family in an organism varied from one to three. Single gene was maintained in most organisms, and three genes are occasionally found in a few species. The duplications of both gene families were observed in cereals and a few other species, thus they were designated as *MAGO1* and *MAGO2*, *Y14a* and *Y14b*. Gene structure of these two families was quite well conserved. *MAGO* genes had three exons in higher plants, four exons in alga, and two to five exons in animals, while *Y14* genes had four exons in higher plants with two exceptions, i.e., three in *Arabidopsis thaliana* and five in *Musa acuminata*, two exons in alga, and two to six exons in animals.

Consistence with the rice draft genome, we isolated two *MAGO* (*OsMAGO1* and *OsMAGO2*) and two *Y14* (*OsY14a* and *OsY14b*) genes from the rice cultivar ‘Zhonghua 10’. Our structural modeling suggested that OsMAGO1 and OsMAGO2 possessed six paralleled β-strands (β1-β6) and three α-helices (α1-α3) arranged as Nt-β1-β2-β3-β4-α1-β5-β6-α2-α3-Ct (Figure S1a and b in File S1). OsY14a had five paralleled β-strands (β1-β5) and two α-helices (α1-α2) in an Nt-β1-α1-β2-β3-α2-β4-β5-Ct arrangement (Figure S1c in File S1), and OsY14b had a similar tertiary structure as OsY14a but with an additional α-helix (α1′) in the N-terminal (Figure S1d in File S1). They are in accordance with the crystal structures of the MAGO and Y14 proteins respectively in *Drosophila* [15], [32] and human [30]. The quality of protein models was assessed and the related parameters are within the range of those accepted for homology based structure models, suggesting the reliable quality estimates of these structures in the present work (Table S4 in File S1). These results suggest a high structural conservation of the MAGO or Y14 proteins across plants and animals, thus hinting a conserved evolutionary pattern of the two protein families.

### Non-Uniformly Slow Evolutionary Rates of the MAGO and Y14 Protein Families

**Figure 1:**
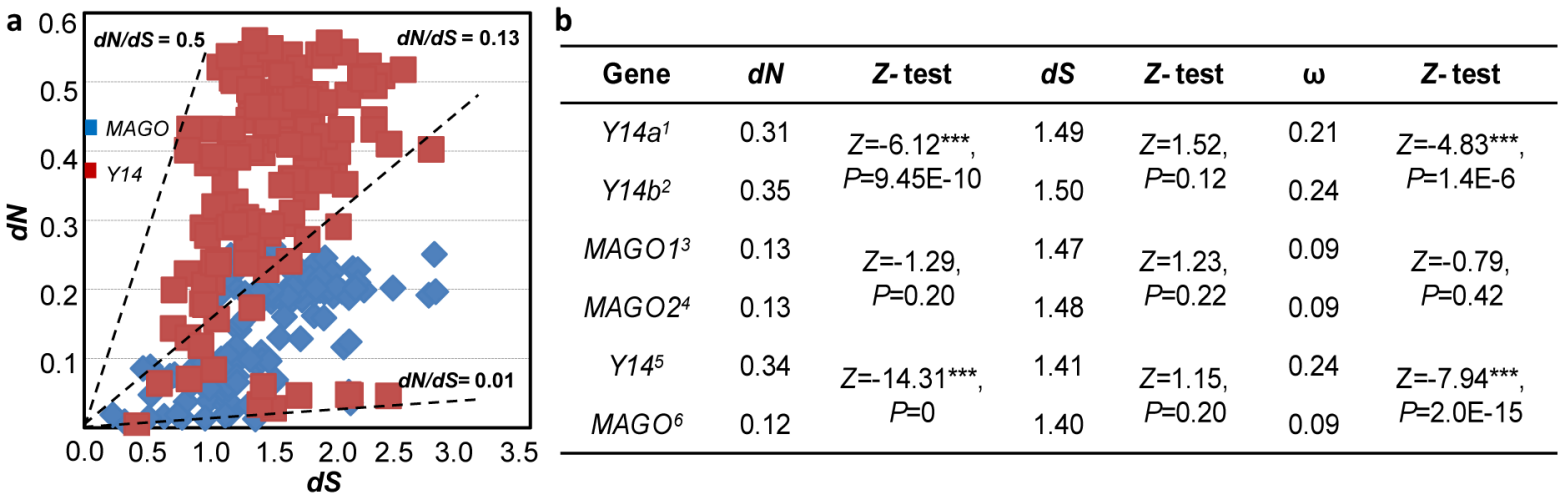
Evolutionary rates and purifying selection of the *MAGO* and *Y14* families. (**a**) Evolutionary rates of the MAGO and Y14 families inferred from all included homologous sequences. *dN*: non-synonymous substitution rate; *dS*: synonymous substitution rate. ω = *dN*/*dS*. (**b**) Comparison of the evolutionary rate. *dN* and *dS* values were determined with different inputs. 1, *Y14* genes excluding *Y14b* copies; 2, *Y14* genes excluding *Y14a* copies; 3, *MAGO* genes excluding *MAGO2* copies. 4, *MAGO* genes excluding *MAGO1* copies; 5, all *Y14* genes and 6, all *MAGO* genes.

To reflect the evolutionary rates, we calculated values of both *dN* (non-synonymous substitution rate) and *dS* (synonymous substitution rate) of the *MAGO* and *Y14* gene families. *dS* varies between the *MAGO* gene family (from 0.09 to 3.14) and the *Y14* gene family (from 0.23 to 2.73), while *dN* ranges from 0.002 to 0.31 for the *MAGO* gene family and from 0.03 to 0.57 for the Y14 family (Figure 1a). Nonetheless, average *dS* values had no significant difference between the *MAGO* and *Y14* families, thus the relative evolutionary rates could be reflected by *dN* values. The average *dN* values were small (0.12 for *MAGO* and 0.34 for *Y14*), and they were significantly different (*Z* = −14.31, *P* = 0) (Figure 1b), suggesting that the two families evolved slowly with the non-uniform evolutionary rates. To evaluate the effect of gene duplication, we used single gene in one organism for each gene family to re-estimate the evolutionary rate. But, no difference was observed between *MAGO1* and *MAGO2* genes within the *MAGO* family (Figure 1b), while significant difference of *dN* was observed between *Y14a* and *Y14b* genes (*Z* = −6.12, *P* = 9.45E-10). In addition, *Y14b* evolved faster than *Y14a* and both were faster than *MAGO* genes (Figure 1b). These results confirmed the differential evolutionary rates of the two families. Consistent to this, MAGO proteins shared 80.2% identity and Y14 proteins had 50.3% identity in amino acid sequences within eukaryotic organisms examined. These results suggest that the two protein families might be under different selection forces.

### Both MAGO and Y14 Protein Families Underwent Different Purifying Selection

The ω (*dN*/*dS*) value was used to reflect the type of selection that a protein might have undergone. The ω values of nearly all proteins ranged between 0.02 and 0.50 in the MAGO and Y14 protein families (Figure 1a), thus indicating that they are basically under a purifying selection. However, the average ω values of the MAGO (0.09) and Y14 family (0.24) had a significant difference (*Z* = −7.94, *P* = 2.0E-15; Figure 1b), suggesting that the two protein families are under different purifying selection. The Y14 family was under relative relaxed purifying selection, while the MAGO family was under stringent purifying selection. Interestingly, the purifying selection of Y14a (ω = 0.21) and Y14b (ω = 0.24) within the Y14 family was significantly different (*Z* = −4.83, *P* = 1.4E-6; Figure 1b). The ancestral sequence reconstruction revealed that *OsY14a* (59.49% identity) was more similar to the pre-duplication ancestor than *OsY14b* (40.65% identity), while *OsMAGO1* (87.95%) and *OsMAGO2* (90.96%) had similar identities with the pre-duplication ancestral sequence.

### The Co-Evolutionary Signal at Both MAGO and Y14 Protein Levels

**Figure 2:**
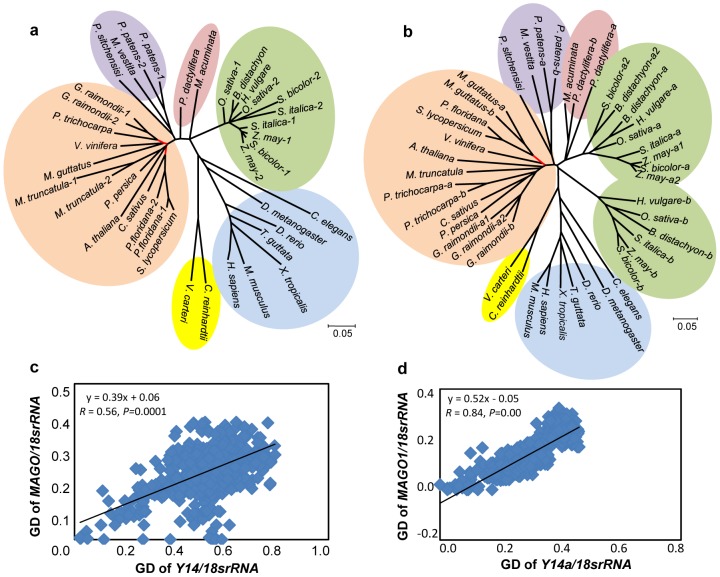
*Tol-MirrorTree* analyses reveal the co-evolution of the MAGO and Y14 families at protein levels. (**a**) Phylogenetic tree of the MAGO protein family. (**b**) Phylogenetic tree of the Y14 family. These ML trees include sequences from algae (yellow), animals (blue), dicots (orange), monocots: grass (green) and others (pink), and gymnosperm and moss (purple). The red lines represent the branches with less than 50% bootstrap in the trees. (**c–d**) Evaluation of tree similarity. Genetic distances (GD) between MAGO and Y14 sequences were corrected by 18s rRNA to avoid a contribution of speciation. (**c**) Similarity of the ML trees with the whole set of sequences shown in **a** and **b**. (**d**) Tree similarity when single sequence for each family in one organism. Similarity evaluation of the ML trees with an inclusion of *MAGO1* and *Y14a* sequences is presented in (**d**). Pearson correlation coefficient is 0.61 (*P* = 0.00) when *MAGO2* and *Y14b* sequences were included.

The molecular phylogeny usually gives the first glimpse of the evolutionary history of the molecule. We therefore constructed the phylogenetic trees of the MAGO and Y14 protein families based on the multiple sequence alignments (Dataset S1 and S2, see Methods). We observed that the duplicated members of the MAGO family were clustered to one group (Figure 2a), while the Y14 family in cereals was represented by two subgroups: Y14a and Y14b (highlighted with green background in Figure 2b). However, the two gene families shared similar topology of phylogenetic trees (highlighted with black lines, Figure 2a and b) indicating that they have a similar evolutionary history. The Pearson correlation coefficient (measure of genetic distance) was estimated in pairwise between *MAGO* and *Y14* trees. 18s rRNA was used to correct the tree similarity due to speciation [52]. This *Tol-MirrorTree* analysis revealed that the correlation coefficient of the *MAGO* and *Y14* trees was 0.56 (*P* = 0.0001) (Figure 2c). Similar results were obtained when one set of duplicated genes were included (*R* = 0.84 or *R* = 0.61, *P* = 0.00; Figure 2d) indicating that the evolution of the *MAGO* family is highly correlated with that of the *Y14* family. As controls, we analyzed the relationship between the LEAFY (LFY) and MAGO or Y14 families. AtLFY, a regulatory factor for floral meristem in *Arabidopsis* [53], and we found that AtLFY and its ortholog in rice (OsLFY) did not interact with MAGO or Y14 proteins from various species (Figure S2a in File S1). Phylogenetic reconstructions revealed that these genes also had a similar tree topology in plants (Dataset S1, S2 and S3; Figure S2b-d in File S1). However, after correction with 18s rRNA, no correlation between MAGO-LFY trees (*R* = 0.20, *P* = 0.06) and Y14-LFY trees (*R* = 0.12, *P* = 0.11) was observed in plants (Figure S2e and f in File S1) suggesting that the LFY family lack co-evolutionary relationship with either MAGO or Y14 family. These analyses, therefore, suggest a potential co-evolution between the MAGO and Y14 protein families.

### The Obligate MAGO-Y14 Heterodimerization Mode Is Plesiomorphic

**Figure 3:**
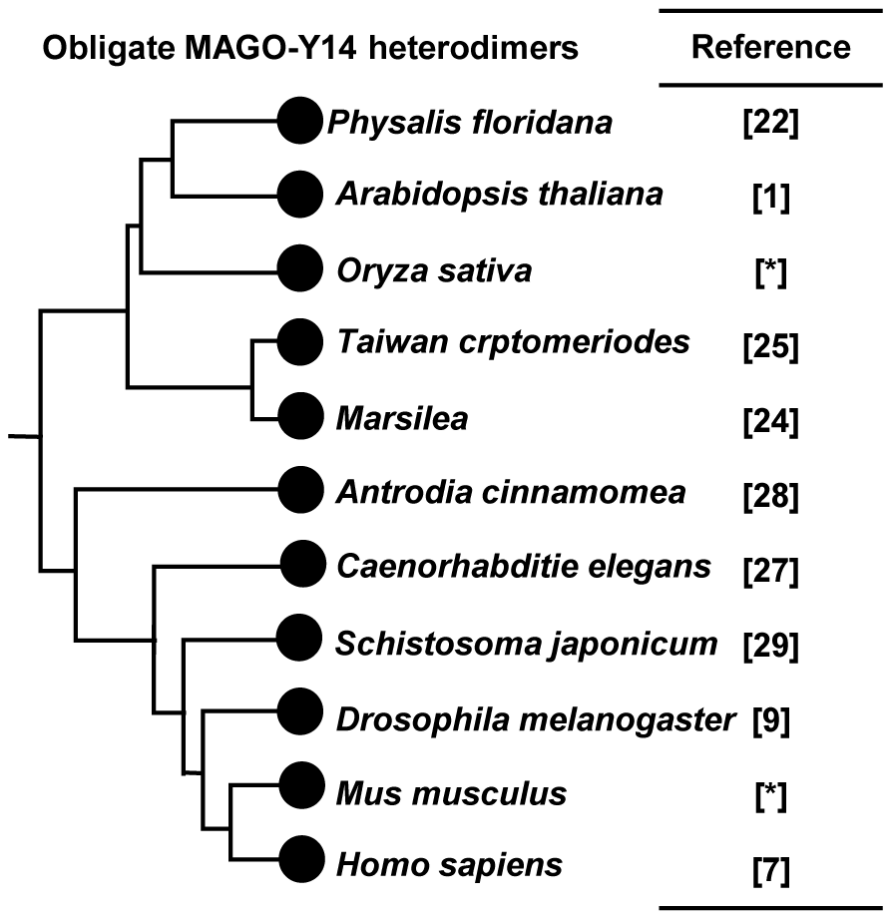
Evolution of the MAGO-Y14 interaction mode. The experimental data from the corresponding references are listed. Star [*] indicates the data from the present work (Figure 4). Filled circles indicate the presence of the MAGO-Y14 interactions that appeared to be a plesiomorphic trait.

**Figure 4:**
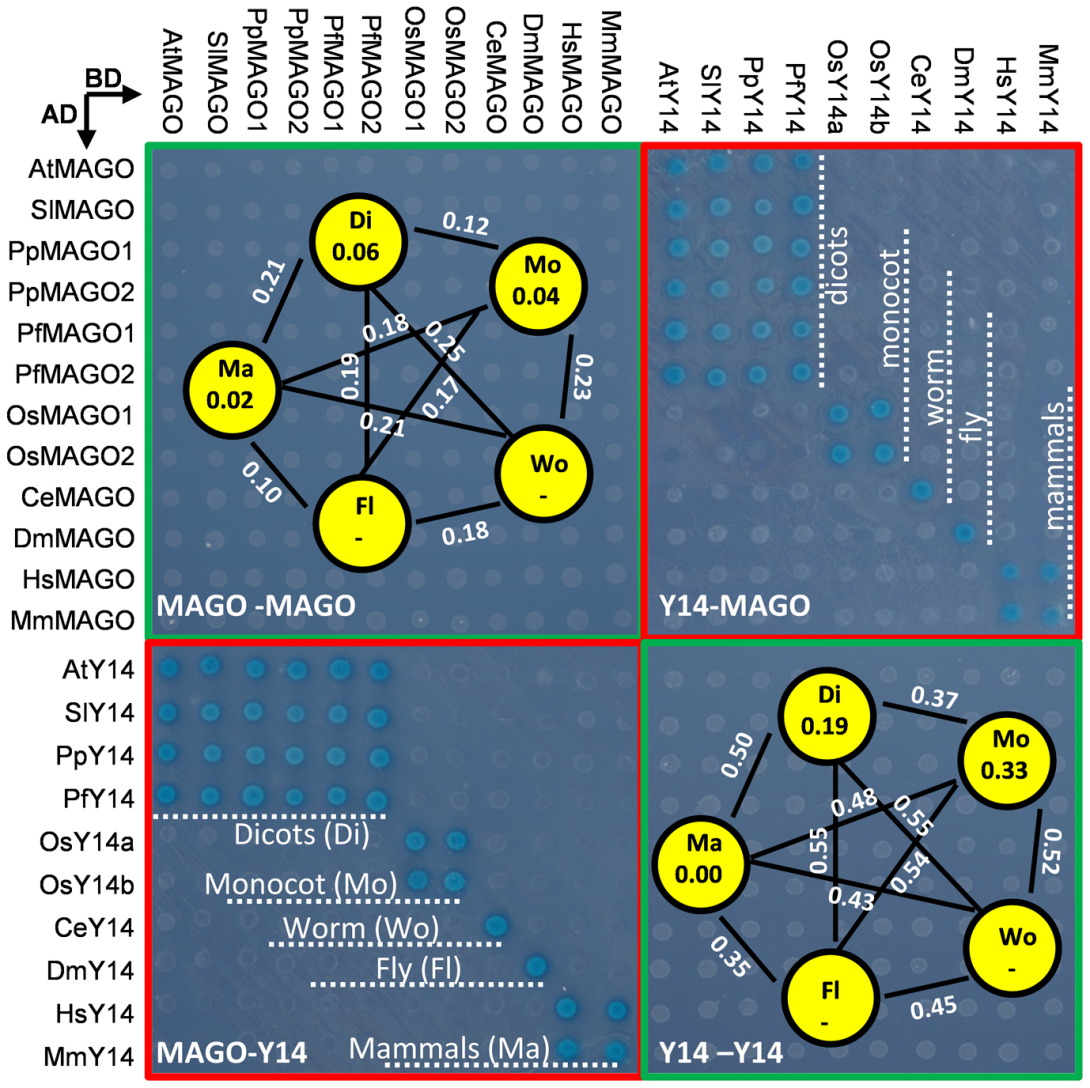
Protein interaction matrices of the MAGO and Y14 proteins from different species. The combination of the bait proteins (BD) and the prey proteins (AD) is indicated. The name of MAGO and Y14 sequences started with the abbreviated name of the species such as *Arabidopsis thaliana* (At), *Solanum lycopersicum* (Sl), *Physalis philadelphica* (Pp), *Physalis floridana* (Pf), *Oryza sativa* (Os), *Caenothabditis elegans* (Ce), *Drosophila melanogaster* (Dm), *Homo sapiens* (Hs) and *Mus musculus* (Mm). The average genetic distance of MAGO and Y14 among dicots (Do), monocots (Mo), worm (Wo), fly (Fl) and mammals (Ma) was given. Dash (-) indicates no data due to single sequence was used in that clade. The survived cells of the same amounts of the co-transformed yeast cells on the highest stringent conditions (SD/Leu-Trp-His-Ade) were subjected to a non-lethal β-galactosidase assay.

The physical interaction of MAGO and Y14 is required for their biochemical and biological roles [14], [19], [31]. To understand the evolutionary history of the interaction, we summarized the available interaction data between MAGO and Y14 proteins in a phylogenetic context (Figure 3). The invariant nature of dimerization implied that this might be a plesiomorphic character present in the last common ancestor of animals and plants. As expected, it was inferred to be the ancestral state in one organism by a formal analysis (not shown). No homodimerization occurred within either MAGO or Y14 families (Figure 3), as experimentally demonstrated in Figure 4, thus suggesting that the heterodimerization is obligate, and the specific mode had been established since their origin in their eukaryotic ancestors, and maintained to be extremely conserved during evolution. To maintain the conserved obligate heterodimerization mode in each eukaryotic species, conservation of their remaining sequences during evolution might play a role. However, the co-evolution of the MAGO and Y14 families is likely essential.

### Functional Barrier of MAGO-Y14 Heterodimerization between Distantly Clades

To further understand the co-evolution of the MAGO and Y14 protein families, we tested the mutual heterodimerization of the protein pairs from various taxon clades (Figure 4). Each bait protein neither could activate *lac*Z and *HIS*3 reporter genes, nor was toxic to yeast cells indicating lack of self-activation of each of them. Next co-transformation analyses of reciprocal combination of bait and prey constructs were conducted. The same amounts of the transformed yeast cells from the SD/-Trp-Leu plates were spotted on the SD/-Trp-Leu-His-Ade plates for all combinations. The results revealed that proteins within either MAGO or Y14 family did not form dimmers (highlighted in green box in Figure 4). Interactions were only detected in some specific combinations between MAGO and Y14 proteins revealed by non-lethal β-galactosidase assay (highlighted in red box in Figure 4) indicating a clade-specific heterodimerization mode of the MAGO and Y14 proteins. Functional barrier (lack of binding of MAGO to Y14 across different clades) between the two protein families was not observed within dicots or the duplicates of rice, while it happened between dicots and monocot (rice). MAGO proteins from worm, fly and mammals did not substitute each other to heterodimerization with any Y14 protein from these clades. However, no functional barrier was observed between mouse and human within mammals. Functional barrier of the MAGO or Y14 protein families between plants and animals was apparent.

We also investigated the relationship of the occurrence of the functional barrier and the genetic distances of the MAGO and Y14 sequences (Figure 4). The average genetic distances within the studied taxon clades were from 0.02 to 0.06 for the MAGO proteins and 0 to 0.33 for the Y14 proteins, while the genetic distance of inter-clades ranged from 0.10 to 0.25 in the MAGO family and from 0.35 to 0.55 in the Y14 family. These results further corroborated the observations of the non-uniform evolutionary rates and different purifying selection of the two protein families. Moreover, the average genetic distances of the inter-clades were significantly larger than these of the intra-clades for both protein families (*P*<0.002). Furthermore, the genetic distance apparently correlated to functional barrier of the inter-clade protein pairs.

The lack of interactions between inter-clade pairs of proteins particularly highlights the co-evolution of the MAGO and Y14 pair to generate a specific mode of interaction, while lack of functional barrier within dicots, rice (between duplicates) and mammals suggests that the co-evolutionary process is slow.

### The Correlated Mutations between the MAGO and Y14 Protein Families

**Figure 5:**
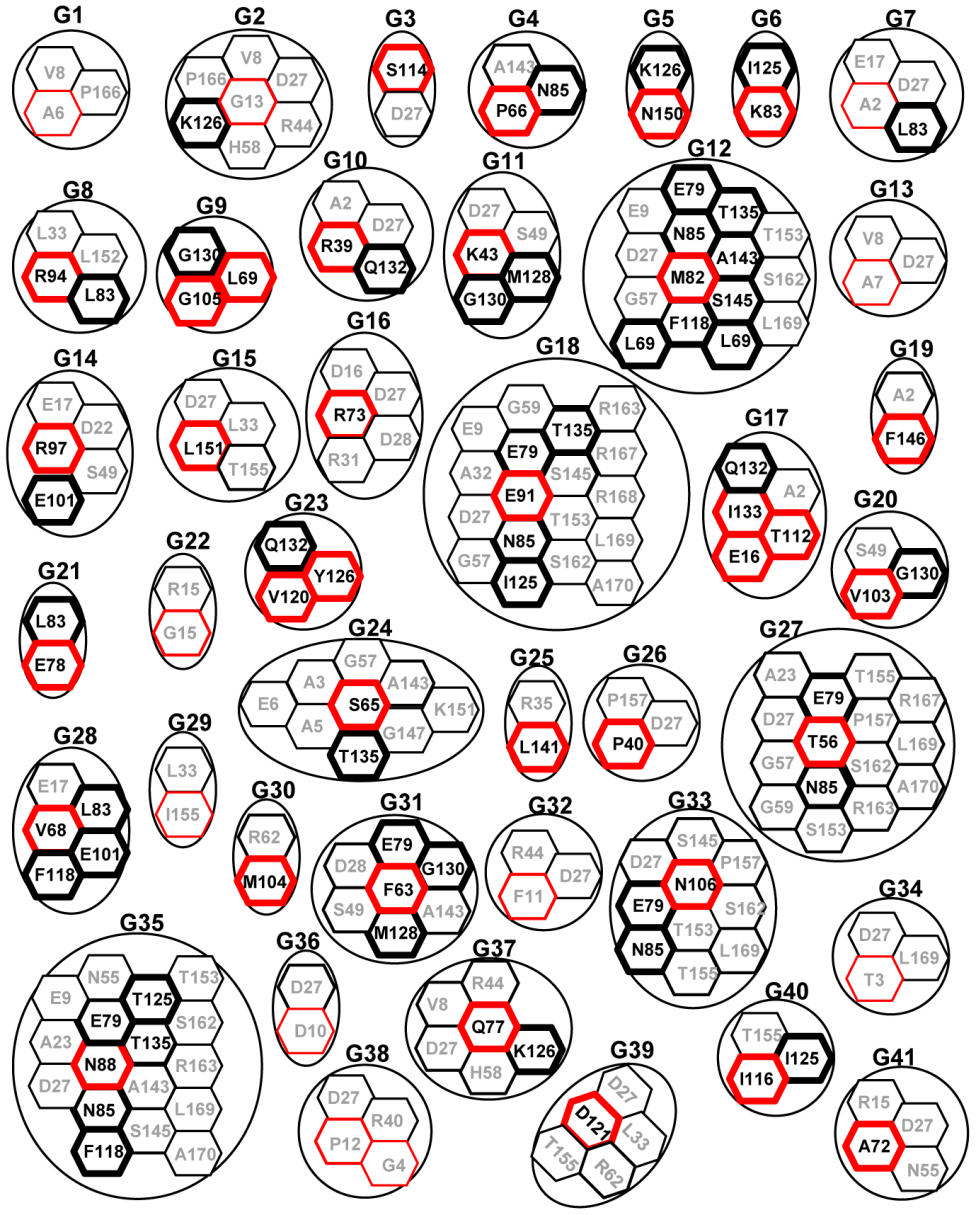
Correlated mutation groups between the MAGO and Y14 families. Groups (G1–G41) of the correlated mutation residues were detected by CAPS. All residues included in one circle are predicted to co-evolve between each other. The correlated mutation residues of the Y14 and MAGO are represented in black and red hexagon, respectively. Functionally or structurally important sites in the MAGO-core or Y14-RBD are in bold box, while others sites are in thin box. The position of the correlated mutational residues in the MAGO and Y14 proteins is referred to as OsMAGO2 and OsY14b, respectively. For the details, see Table S5 in File S1.

To understand their co-evolution at molecular levels, the correlated mutational residues in the two families were therefore predicted by the co-evolution analysis using protein sequences (CAPS). Using Pfam program, we predicted that MAGO proteins contain three regions including N-terminal, MAGO-core (16-158) and C-terminal, while Y14 proteins consist of N-terminal, Y14-RNA binding domain (RBD) (69-139) and C-terminal (Table S5 in File S1). In CAPS, we identified 41 putative groups of the correlated mutational residues between the MAGO and Y14 protein families. They included 97 unique residues (46 in the MAGO family and 51 in the Y14 family), and the residue position was referred as to OsMAGO2 and OsY14b, respectively (Figure 5; Table S5 in File S1). The mean variance for the amino acid transition in each group of co-evolution (Mean Dc) ranged between 2.11 and 11.69, whereas the mean correlation (Mean ρ) varied between 0.34 and 0.68 with the *P*-score<0.05 (Table S5 in File S1) thus supporting their correlations.

Thirty-six residues of the MAGO family (36/46 = 78.3%) were identified in the MAGO-core region that is involved in protein-protein interaction with Y14 [15]; while 12 of the 51 residues (23.5%) in Y14 were identified in the RBD, a domain for RNA binding [15], [30]. However, if the ratio of the correlated residues to the length of the protein or the domain is considered, we found that 25.2% (36/143 in the MAGO-core) and 16.9% (12/71 in the Y14-RBD) of functionally important sites might involve the co-evolutionary process. 41.7% residues of MAGO proteins (10/24) and 41.5% residues of Y14 proteins (36/94) in their own N- and C-terminals were also involved in co-evolution. Therefore, the correlated mutational sites were not enriched in their functional domains. Nonetheless, 22 (53.7%) groups were observed to contain both functional domains sites of MAGO and Y14, and 11 (26.8%) contained one of them, while 8 groups did not fall into the MAGO-core and Y14-RBD. It should be noted that 17 out of 36 correlated resides in the MAGO-core and 2 out of 12 in the Y14-RBD were conserved, and others were changed; accordingly, 16 of 41 groups were not changed, only 14 out of 25 groups that were altered had happened co-mutations between the MAGO and Y14 families (Figure S3 in File S1). Therefore, these co-altered residues and the 14 correlated groups might be essential in the co-evolution of the MAGO and Y14 protein families.

### Characterization of the Clade-Specific Residues Further Supports the Co-Evolution of the MAGO and Y14 families

**Figure 6:**
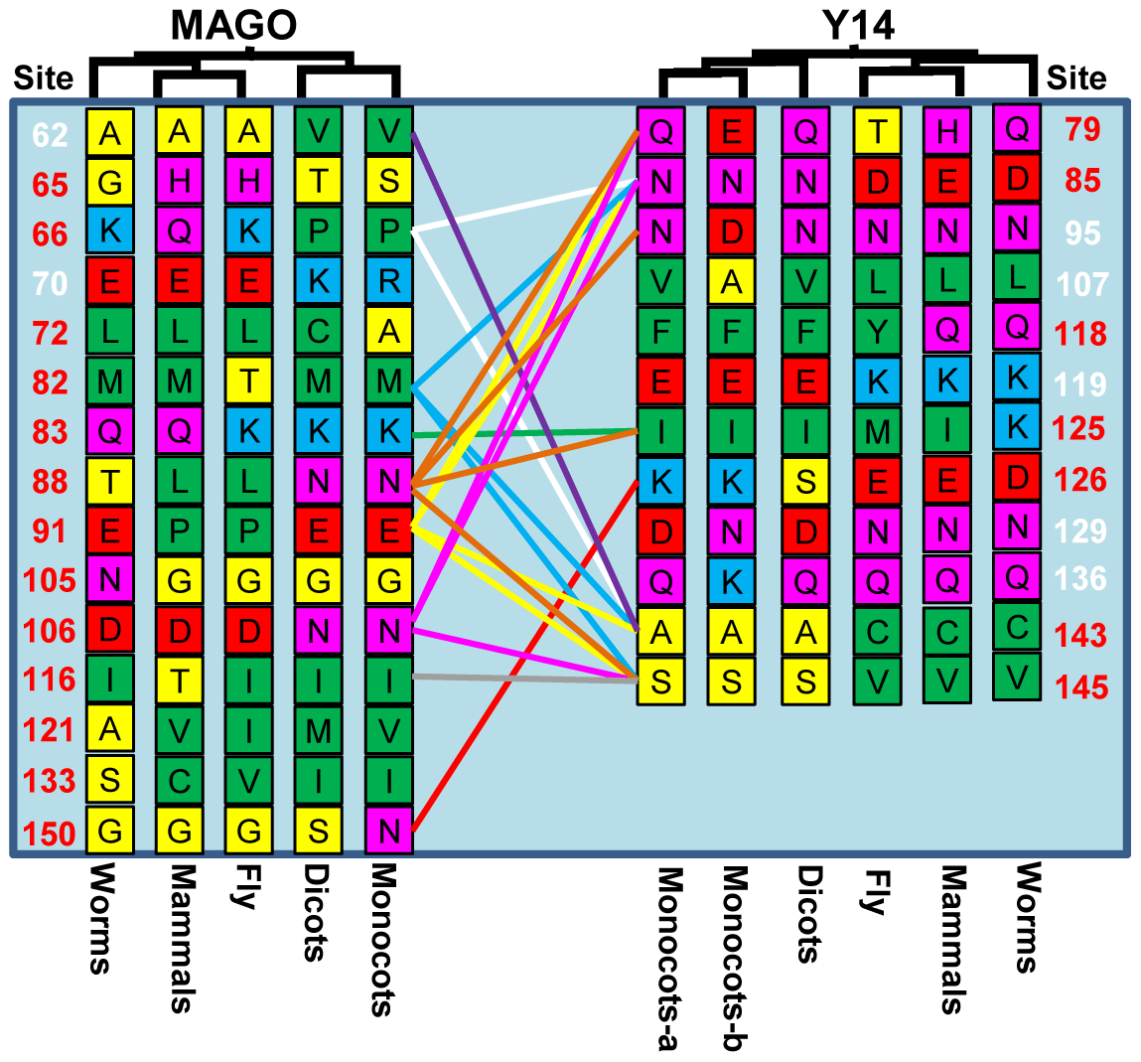
The clade-specific sites in the MAGO and Y14 families. The clade-specific amino acids are arranged that the small nonpolar residues (G, A, S and T) are highlighted in yellow, the hydrophobic residues (C, V, I, L, P, F, Y, M and W) in green, the polar residues (N, Q and H) in magenta, the negatively charged residues (D and E) in red, and the positively charged residues (K and R) in blue. The residues between the two protein families that were predicted to be correlated mutation groups in CAPS were connected by color lines. The black, red, green, blue, yellow, purple, pink, orange and gray line respectively represents the G4, G5, G6, G12, G18, G24, G33, G35 and G40 (Figure 5; Table S5 in File S1). The given position of the residues in the MAGO and Y14 families corresponds to OsMAGO2 and OsY14b, respectively. The red numbers showed that these sites were predicted to be co-evolved in CAPS, the white was not.

CAPS is prone to false positives. In order to narrow down the number of the co-evolution groups and residues, we searched the clade-specific mutated residues based on the MSA (Dataset S1 and S2; Figure 6). We found altogether 15 residues in the MAGO family and 12 residues in the Y14 family were involved in the clade-specific features among dicots, monocots, worms, flies, and mammals. Among these clade-specific residues, 12 from the MAGO family and 7 from the Y14 family were overlapped with the predicted co-mutational residues and covered the nine co-evolved groups (G4, G5, G6, G12, G18, G24, G33, G35 and G40) between the two protein families (Table S5 and Figure S3 in File S1). These residues and groups respectively occupied 19.59% (19/97) and 21.95% (9/41) of the predicted co-evolved residues and groups in CAPS. In particularly, we found that the residues in positions 72 and 150 in MAGO and 126 in Y14 were clade-specific between monocots and dicots (Figure 6). Thus, the clade-specific residues that predicted to be co-evolved in CAPS might play crucial roles in the co-evolution of the MAGO and Y14 families.

### The Co-Evolved or Clade-Specific Residues Largely Fall in the MAGO-Y14 Minimal Interaction Domains

**Figure 7:**
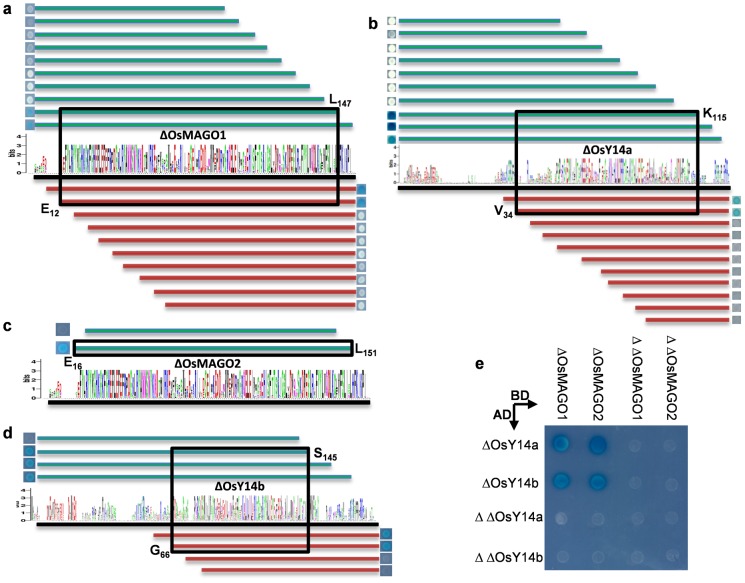
Determination of minimal interaction domains (MIDs) of rice MAGO and Y14 in yeast. (**a**) The MID of ΔOsMAGO1 to interact with OsY14a. (**b**) The MID of ΔOsY14a to interact with OsMAGO1. (**c**) The MID of ΔOsMAGO2 to interact with OsY14b. (**d**) The MID of ΔOsY14b to interact with OsMAGO2. The deletion versions of the proteins were generated as indicated in **a**-**d**. The defined MIDs of the OsMAGO1/2 and OsY14a/b are boxed. The log for each protein family is presented. (**e**) Confirmation of the MID in yeast. Further deleted versions (ΔΔ) did not maintain the capability to interact, while the MID (Δ) formed heterodimer similar to their native proteins. Non-lethal β-galactosidase assays were performed suggest their interactions.

To further understand the role of the co-evolution of the MAGO and Y14 families in their heterodimerizations, we determined the minimal interaction domain (MID) for their interactions. We performed a large set of deletions on OsMAGO1/2 and Y14a/b and tested their consequence in heterodimerization capability. For example, we made series deletions of OsMAGO1 from each terminal, and then tested the interaction capability of the resulted version protein with the intact OsY14a; when the interaction was completely abolished, then the minimal interaction region was roughly determined (Figure 7a). Following this strategy, we found that ΔOsMAGO1 (E_12_-L_147_) and ΔY14a (V_34_-K_115_) corresponding to ΔOsMAGO2 (E_16_-L_151_) and ΔOsY14b (G_66_-S_145_) were the MIDs for these proteins (Figure 7a-d), and these resulted versions still maintained the heterodimerization capability (Figure 7e). However, further deletions ΔΔOsMAGO1 (E_12_-F_140_ and V_19_-L_147_), ΔΔOsMAGO2 (E_16_-F_144_ and V_23_-L_151_), ΔΔOsY14a (V_34_-P_113_) and ΔΔOsY14b (G_66_-V_138_ and G_73_-S_145_) completely abolished their interactions (Figure 7e). In addition, structural modeling revealed that ΔOsMAGO1 and ΔOsMAGO2 had a similar structure as OsMAGO1 and OsMAGO2, while ΔOsY14a and ΔOsY14b featured the same structure to OsY14a (Figure S1 in File S1). Thus, these deleted protein versions of ΔOsMAGO1 (E_12_-L_147_), ΔOsMAGO2 (E_16_-L_151_), ΔY14a (V_34_-K_115_) and ΔOsY14b (G_66_-S_145_) are the minimal and core regions for the rice MAGO and Y14 interactions. The interaction interface of the MAGO-Y14 was revealed as the parts of the MAGO-core and Y14-RBD based the MAGO-Y14 structure (Figure S4 in File S1). Thus the defined MIDs in yeast fully covered the interaction interface of the MAGO and Y14 proteins.

Compared the Pfam functional domains of the MAGO and Y14 proteins (Table S5 in File S1) with their MIDs (Figure 7) and the interaction interface (Figure S4 in File S1), we found that they largely overlapped. 35 residues of the 46 correlated mutational residues in MAGO (76.1%) fell in to in the MID; while 14 of the 51 residues (27.5%) in Y14 located in the MID. Nearly all the clade-specific residues of the MAGO and Y14 proteins located in their own MID. These residues randomly occurred on the helixes, sheets and random coils of the two proteins (Figure S4 in File S1), while 33% (Y14) and 47% (MAGO) of the correlation sites in their functional domains, and 42% (Y14) and 47% (MAGO) of the clade-specific residues located in the interaction interface. Interestingly, a substantial proportion (around 50%) of the correlated mutation residues and 8 correlated mutational groups (about 20%) were found to be outside of the MID and functional domains of the MAGO and Y14 proteins. Moreover, five correlated residues were clade-specific in the interaction interference of the MAGO-core, while the clade-specific residues and the predicted co-evolved sites in the interaction interface did not overlap in the Y14-RBD (Figure S4 in File S1), reflecting an additional role of the co-evolution. Nonetheless, our findings suggested that the co-evolution might be required for the maintenance of obligate heterodimerization mode between MAGO and Y14 in one organism during evolution.

### Crucial Roles of the Co-Evolution in the Maintenance of the MAGO-Y14 Heterodimerization Mode

**Figure 8:**
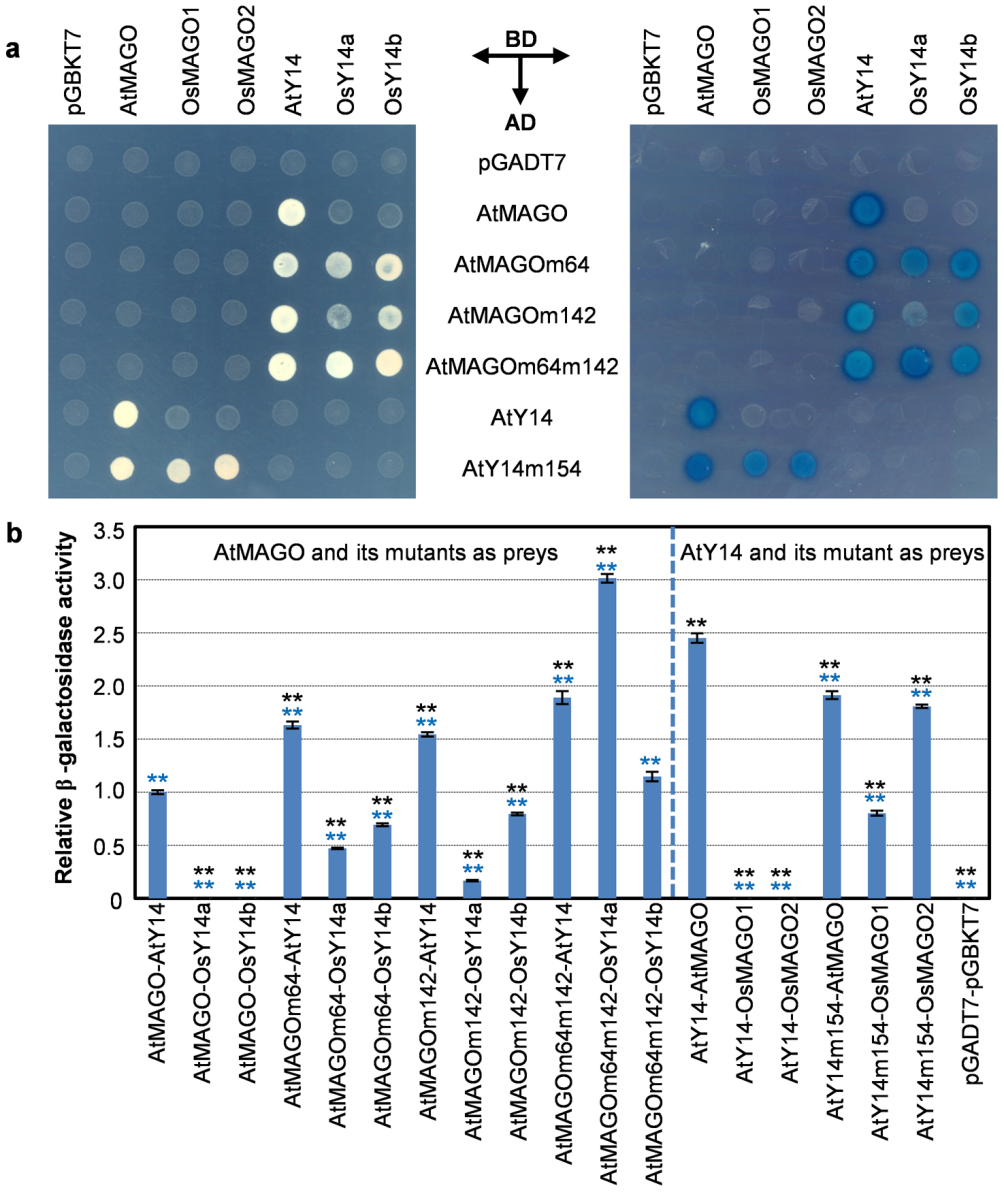
Crucial roles of the clade-specific residues in the MAGO-Y14 heterodimerization. (**a**) Protein-protein interactions in yeast. The combination of the bait proteins (BD, horizontal arrows) and the prey proteins (AD, vertical arrow) is indicated. Left panel: The growth of the same amounts of the co-transformed yeast cells on the highest stringent conditions of the SD/Leu-Trp-His-Ade plates. Right panel: The result of the non-lethal β-galactosidase assay. The clade-specific residues 64 and 142 of AtMAGO and the residue 154 of AtY14 in *Arabidopsis* (a dicot) were mutated to the corresponding sites in the rice proteins, the representatives from monocots (Figure 6), and the resultant proteins were AtMAGOm64, AtMAGO142, AtMAGOm64m142 and AtY14m154. The combinations of the BD proteins and pGADT7 or the AD proteins and pGBKT7 were included as negative controls. (**b**) Quantification of the heterodimerization strength. The relative β-galactosidase activity was normalized with the interaction strength of AtMAGO -AtY14 (AtMAGO as prey). The combination of the pGADT7 and pGBKT7 empty vectors was included as a negative control. The experiments were repeated three times. The average enzyme activity and the standard deviation are presented. The significance of the strength difference between interactions was evaluated using two-tailed *t*-tests (*P* = 0.000). The black stars (**) indicate the comparison to AtMAGO -AtY14 (AtMAGO as prey), while the blue stars show the comparison to AtY14-AtMAGO (AtY14 as prey).

To confirm the above notion, we performed the site-directed mutagenesis analysis. The three correlated or clade-specific residues in the two proteins could distinguish the sequences between dicots and monocots (Figure 6). We mutated these sites in *Arabidopsis* and evaluated the effects of the mutagenesis on their heterodimerizations in yeast. When the *Arabidopsis*-specific residues in AtMAGO (C_64_ and S_142_) were mutated into the residues (A and N) in rice, three mutated proteins were synthesized as AtMAGOm64, AtMAGOm142 and AtMAGOm64m142. Each single-site mutation (AtMAGOm64 or AtMAGOm142) and the double-site mutational proteins AtMAGOm64m142 became to heterodimerize with rice Y14 proteins (OsY14a and OsY14b); when the *Arabidopsis*-specific residue in AtY14 (S_154_) was mutated into the residue (K) of rice Y14 proteins, the mutated AtY14m154 interacted with OsMAGO proteins (OsMAGO1 and OsMAGO2). Moreover, all mutated proteins still kept the capability and pattern to heterodimerize with the native *Arabidopsis* proteins (Figure 8a); however, the interaction strength seemed to be different. We next quantified their interaction strengths. In a contrast with the ‘functional barrier’ between rice proteins and *Arabidopsis* protein, all site-mutated AtMAGO proteins as preys obtained a substantial affinity with rice Y14 proteins, and the interaction of the double-site mutation with OsY14a was even significantly stronger than the native AtMAGO-AtY14 interaction (*P* = 0.000); while the mutated AtY14 as prey showed a slight reduction in interaction with AtMAGO, and gained a moderate interaction strength with rice MAGO proteins in comparison to the AtY14-AtMAGO interaction (Figure 8b), suggesting the crucial role of these sites in the MAGO and Y14 heterodimerization. Unexpectedly, the mutated *Arabidopsis* proteins did not completely abolish their heterodimerization capability with the native *Arabidopsis* proteins; on the contrary, all mutated AtMAGO proteins had an unexpected dramatic increase in the capability to interact with AtY14 (*P* = 0.000), hinting a role of other correlated sites in the MAGO-Y14 interaction and a bias discrepancy in amino-acid usage between dicots and monocots. Therefore, our results reveal the crucial role of the correlated and clade-specific residues in the maintenance of the MAGO-Y14 heterodimerization mode during evolution.

## Discussion

As the core components of the EJC, MAGO and Y14 play essential biochemical and developmental roles. In this study we showed that the two protein families formed obligate MAGO-Y14 heterodimer and co-evolved slowly under different purifying selection since their origin in eukaryotes.

### The Origin of the Co-Evolution of the MAGO and Y14 Protein Families

The inter-protein co-evolutionary signal should locate in their primary sequences, thus various analyses based on multiple sequence alignments were performed. The congruence of phylogenetic topology of the two protein families provides the first indicator to distinguish whether they were under the process of co-evolution [35]–[38]. Our *Tol*-*MirrorTree* analyses suggested that the MAGO and Y14 protein families have co-evolved. 41 groups of significantly correlated mutation residues were identified between the MAGO and Y14 protein families. In the MAGO family, these residues (76%) were mostly localized in the MAGO-Y14 minimal interaction domains (MIDs), and around 28% of the correlated mutational residues of the Y14 family located in the MID and 33–47% mutational residues (including clade-specific residues) located in interaction interface of MAGO-Y14 complex. Moreover, the clade-specific residues in the MSA were found to cover 20% the predicated correlated mutation residues and groups. These observations hint that co-evolution might play a vital role in the maintenance of the MAGO-Y14 heterodimerization mode.

Our ancestral state analysis revealed that MAGO and Y14 evolved obligate heterodimerization pattern since their origin in eukaryotes, as experimentally shown in *Drosophila* [15], human [30], *Arabidopsis* [12], *Physalis* [22], rice and others (in the present work). To maintain this interaction mode, mutations in one protein could lead to alterations in another protein in the complex to generate compensatory changes. This ensures that they fit each other to make the complex stable and adaptable. However, the obligate interactions can also be maintained through remaining conservation in sequences. Co-evolution of the protein pair that interacts should be ultimately proved by functional barrier experiments [41]. This notion was well evident from the observations that inter-protein co-evolution of the sex-determining proteins in nematode Fem-3 and Tra-2 form functional barriers in a strictly species-dependent manner [39]. The co-evolution of proliferating cell nuclear antigen (PCNA)-partner networks can form functional barriers between fungal species [40]. In our large scale yeast two hybrid analyses, the lack of interactions between inter-clade pairs of MAGO and Y14 highlights the role of the co-evolution in generating a specific mode of interaction of MAGO and Y14 pair. The consequence of the site-directed mutagenesis of the clade-specific residues in dicot (*Arabidopsis*) was further corroborated this statement.

MAGO can completely mask the RNA binding domain [7], [9], [26] through its MAGO-core. And indeed we found that the MID and interaction interface of Y14 basically overlap with the RBD domain of this protein. However, a substantial proportion of the correlated mutational residues (around 24% for MAGO and 72% for Y14) located outside of the functional domains of these proteins. Moreover, the clade-specific resides and the predicted co-evolved sites in the interaction interface did not overlap in the Y14-RBD (Figure S4 in File S1). Thus, the co-evolution might play an additional role. The correlated mutational sites (including the clade-specific residues) outside of the MIDs or interaction interface might facilitate the heterodimerization of MAGO and Y14 and optimize its structure for a higher order complex formation or these residues might be involved in the maintenance of the conserved protein structure. These notions were supported by our observations of the highly conserved structures of these proteins across animals and plants during evolution.

Undoubtedly, the compensatory changes in the protein pair contribute to co-evolution of the MAGO and Y14 protein families. Lack of the heterodimerization between the inter-clade protein pairs correlates to the genetic distances, which verifies the co-evolution of the MAGO and Y14 protein families. Nonetheless, functional substitution of MAGO and Y14 for heterodimeriation within each clade of dicots (*Arabidopsis*, tomato and two *Physalis* species), rice (between duplicates) and mammals (mice and human) largely agree that the co-evolutionary process is extremely slow. The two proteins that form obligate heterodimer might co-evolve; however, no concrete evidence was observed. In the present work, we found the internal force for the co-evolution of the MAGO and Y14 protein families. In addition, *MAGO* and *Y14* genes have overlapping expression patterns and their proteins are co-localized in animals and *Arabidopsis* [1], [6], [26]. These also act as external forces [35]–[37] contributing to the co-evolution of the MAGO and Y14 families.

### The Co-Evolved MAGO-Y14 Represents a New Obligate Heterodimerization Mode

Obligate protein heterodimerization is a well-studied molecular interaction, and the two proteins in the complex are known to have shared functions. This was evident from the observations that floral B-class functional heterodimer GLOBOSA (GLO)- DEFICIENS (DEF) evolved for organ identity specification of the petal and stamen in flowering plants [54], [55], and that the archaeal Alba1-Alba2 heterodimer has an effect on DNA packaging [56]. GLO and DEF are MADS-box regulatory paralogs [57], while Alba1 and Alba2 are sequence closely related chromatin proteins [56]. The obligate heterodimerization in the two systems evolved from the homodimerization and the process in still ongoing [54], [56]. However, MAGO and Y14 belong to distinct gene families encoding RNA binding proteins without any homology that strictly form obligate heterodimer in a eukaryotic organism. Thus, the MAGO-Y14 represents a new type of the obligate heterodimer system.

Obligate heterodimer may function in the same functional pathways, like GLO-DEF in floral development [54], [55] and Alba1-Alba2 on DNA packaging [56]. This notion is supported by the previous observations in animals that either *MAGO* or *Y14* is required for *oskar* mRNA transport [16]–[18], and mutation in any genes causes similar development defects [14], [18]–[20], [27], [31]. This functional constraint, in turn, guarantees their co-evolution.

### Functional Implications of the Co-Evolution of the MAGO and Y14 Families

Co-evolution has usually been found in systems that must evolve quickly or when proteins acquire new functions while keeping the interactions between the involved partners [38]–[40], [58]. Moreover, the rapidly co-evolving proteins usually show similar and correlated evolutionary rates [37], [58], [59] and are generally associated with similar selection forces [58], [60], [61]. In our study, we found that the MAGO and Y14 protein families evolved slowly with non-uniform evolutionary rates. This observation is reinforced by different genetic distances of the two protein families. Proteins with a greater fraction of amino acid residues playing an essential role will, on the whole, evolve slower than those with a small ratio of such crucial residues [62], [63]. In line with this notion, MAGO have more residues (136/160 = 85%) participate in their interaction than Y14 (80/160 = 50%). To maintain the stability of their interacting structures, more sites in MAGO (85%) will be constrained in the process of evolution than Y14 (50%), thus Y14 may be faster than MAGO in evolution. The rapid divergence of the Y14 family may serve as a major driving force for the evolution, while the slow divergence of the MAGO family contributes to the conservation, hinting that they might encounter different selection forces during their co-evolution. Further selection analyses indeed revealed they were under significantly different purifying selection. Thus, our data shows that the MAGO-Y14 system represents a particular and novel co-evolutionary selection process of obligate interaction of protein pairs in eukaryotes.

Functional implications of this molecular co-evolution are important as they are essential to optimize physiological performance and reproductive fitness of organisms [64]. The rapid co-evolution of interacting proteins can form reproductive barriers between organisms as a result of hybrid network incompatibility, and thus can act as a driving force in promoting and fixing speciation [39], [40], [58]. MAGO and Y14 proteins are largely involved in embryogenesis and gametophyte development in both animals and plants as discussed above, thus alteration in any of them might affect the fitness of the eukaryotic organisms. The MAGO and Y14 proteins show slow co-evolution that is required for the maintenance of the proper obligate heterodimer structure that functions as a core part of the fundamental EJC machinery to precisely monitor and regulate gene expression in the development of eukaryotic organisms [1]–[6], thus acting as a strong force in guaranteeing their fitness during evolution.

## Conclusions

Our work reveals the evolutionary patterns of the *MAGO* and *Y14* gene families, and reports the origin, slow but non-uniform divergence, and co-evolution of the two protein families. As a major driving force for their co-evolution, they are obligated to form the MAGO-Y14 heterodimer in a eukaryotic organism, and the obligate interaction mode is a plesiomorphic trait originated in the eukaryotes. Our work, therefore, identified a novel system to understand the co-evolution of protein families. Moreover, we also found that gene duplications had occurred during evolution and particularly at least two copies of each gene family were maintained in cereals. This observation might reflect an adaptive role of these EJC genes in the cereal evolution. Thus, their functional divergence, developmental role in cereals and the conservation between plants and animals are potential interests to be studied next.

## Supporting Information

Dataset S1 The MSA of the MAGO family analyzed in the present work.
(TXT)

Dataset S2 The MSA of the Y14 family analyzed in the present work.
(TXT)

Dataset S3 The MSA of the plant LFY family analyzed in the present work.
(TXT)

File S1 Figure S1–S4, and Table S1–S5.
(PDF)
