# Supplementary material for: Slow Co-Evolution of the MAGO and Y14 Protein Families Is Required for the Maintenance of Their Obligate Heterodimerization Mode
Source: PLoS One. 2014 Jan 8;9(1):e84842. doi: 10.1371/journal.pone.0084842 (PMC3885619; doi:10.1371/journal.pone.0084842)
Supplement: File S1 — Figure S1–S4, and Table S1–S5. (PDF) [file pone.0084842.s004.pdf]

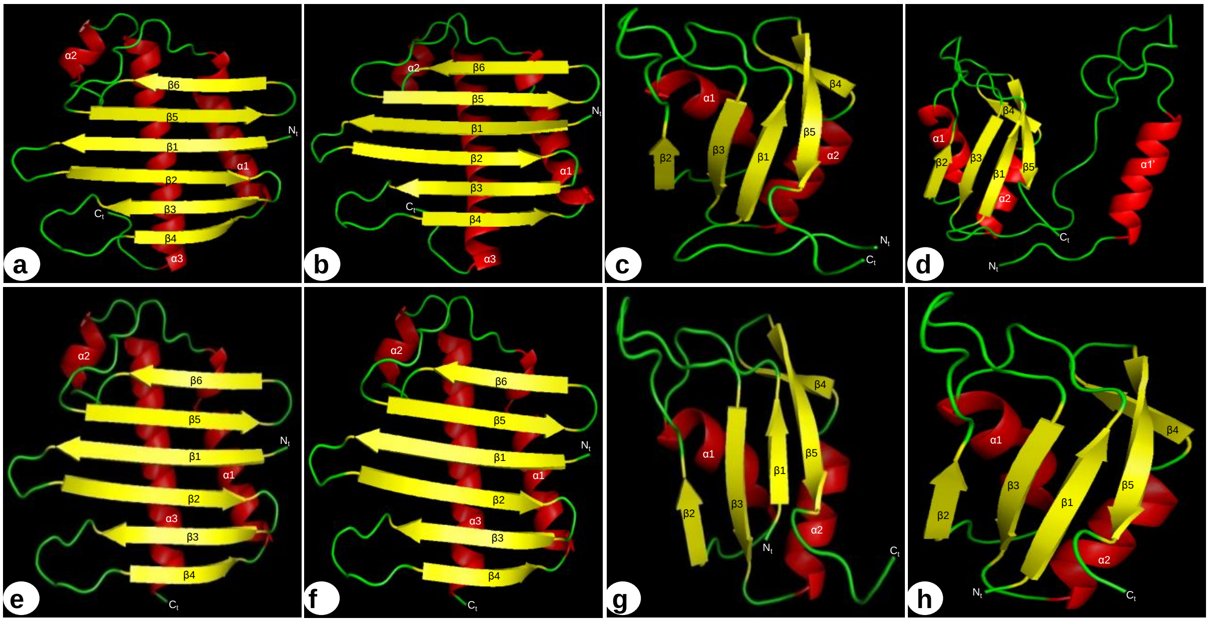

Figure S1. Predictions of tertiary structure of the rice MAGO and Y14 proteins.
(a) and **(b)** Tertiary structure prediction of OsMAGO1 and OsMAGO2. **(c)** and **(d)** Tertiary structure prediction of OsY14a and OsY14b. **(e-h)** The predicted tertiary structure of the minimal interaction domains (MIDs) of the rice MAGO and Y14 proteins. The MIDs were determined by yeast two-hybrid system (Figure 7). **(e)** ΔOsMAGO1 (E_{12} to L_{147}) and **(f)** ΔOsMAGO2 (E_{16} to L_{151}) have a similar structure to these of the native proteins in **a** and **b**. **(g)** ΔOsY14a (V_{34} to K_{115}) and **(h)** ΔOsY14b (G_{66} to K_{145}) feature the same structure to OsY14a in **c**. Ct and Nt: C- and N-terminal; α and β: α-helix and β-sheet.

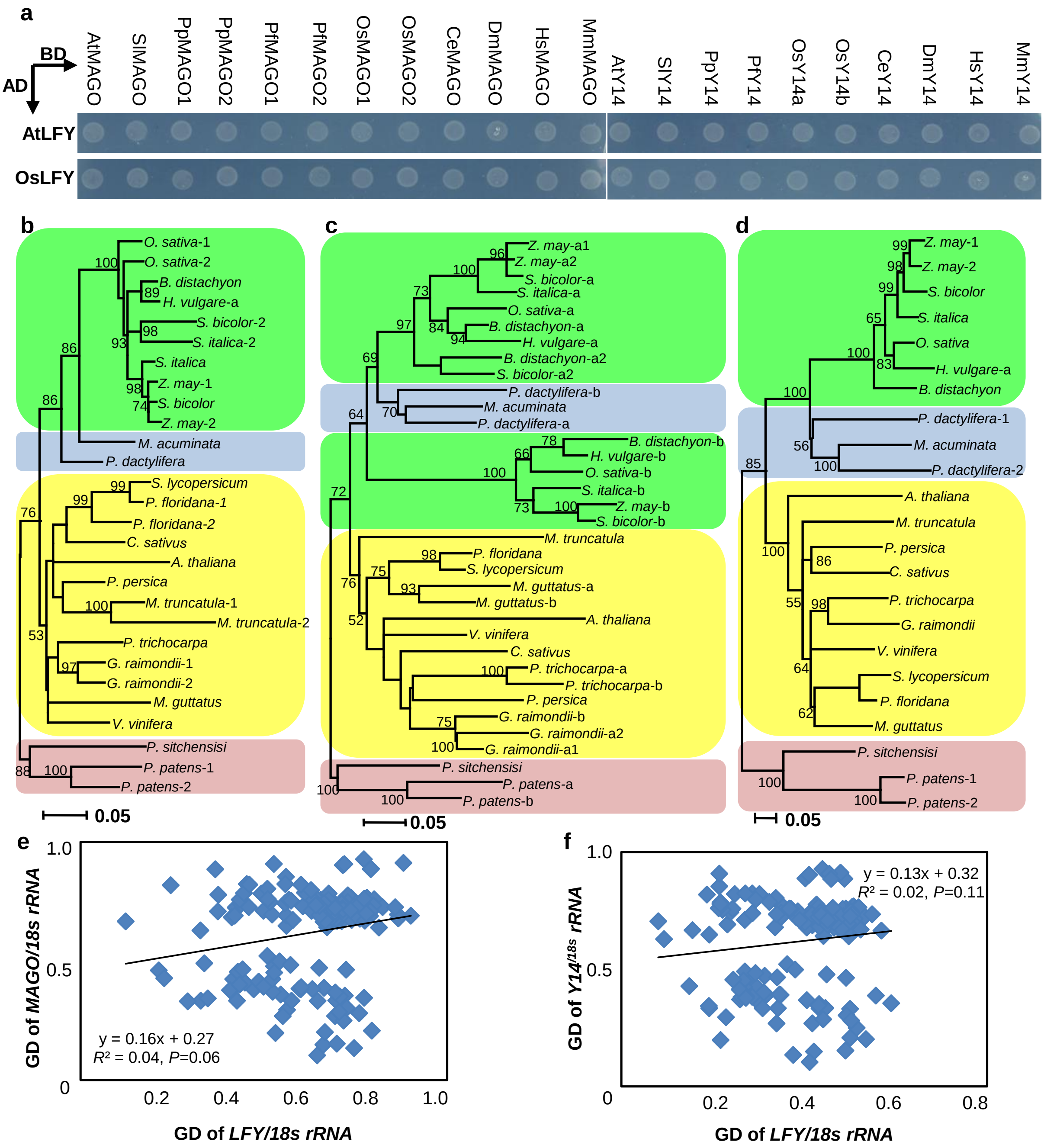

Figure S2. The LFY family did not co-evolve with either MAGO or Y14 family in plants.
(a) Interactions of LFY with either MAGO or Y14 proteins. The AtLFY and OsLFY were included as preys (AD), while the MAGO and Y14 proteins are indicated as baits (BD). The co-transformed yeast cells harboring the BD and AD constructs were selected on the SD/-Leu/-Trp/-His/-Ade plates. No cell growth indicated that AtLEY and OsLFY did not interact with either MAGO or Y14 proteins. **(b)** Phylogenetic tree of the MAGO family in plants. **(c)** Phylogenetic tree of the Y14 family in plants. **(d)** Phylogenetic tree of the LFY family in plants. These ML trees include sequences from dicots (yellow), monocots (cereals in green and others in blue), and gymnosperm and moss (purple). **(e-f)** Evaluation of tree similarity. Genetic distances (GD) between the MAGO and Y14 with the LFY sequences were corrected by 18s rRNA to avoid a contribution of speciation. **(e)** Tree similarity of the MAGO and LFY families. **(f)** Tree similarity of the Y14 and LFY families. No correlation between the LFY and MAGO trees (R = 0.19; P = 0.06) or between the LFY and Y14 trees (R = 0.12; P = 0.11) indicates no co-evolution of the LFY family with either MAGO or Y14 family.

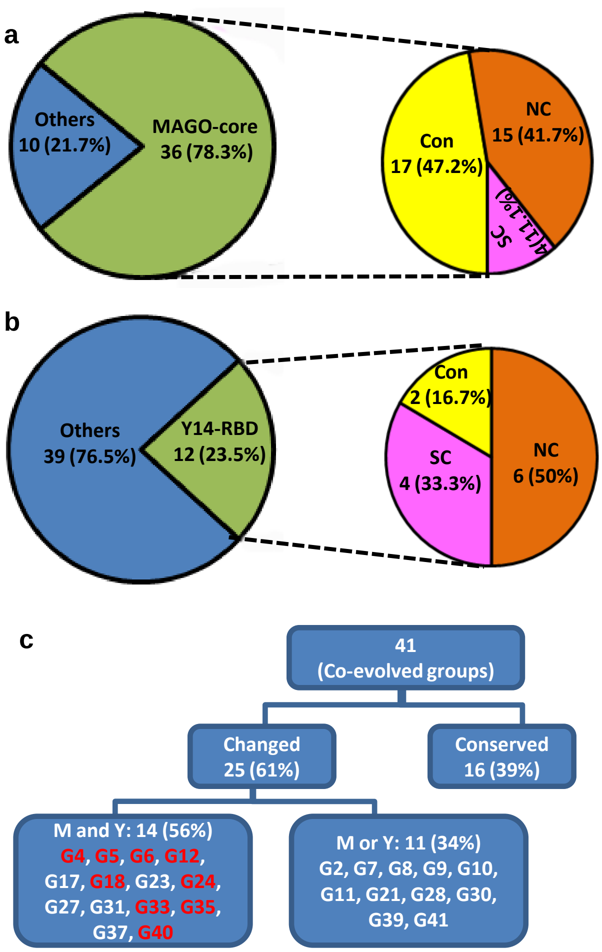

Figure S3. Distribution of the co-evolved residues and groups between the MAGO and Y14 families.

(**a**) The co-evolved residues in the MAGO family. (**b**) The co-evolved residues in the Y14 family. In each functional domain (MAGO-core or Y14-RBD), NC represents the sites with non-synonymous changes; SC represents the sites with synonymous changes; the conserved (Con) stands for the sites unchanged. The 'Others' includes the N- and C- terminals of the proteins. The number of the co-mutational residues is given out of the bracket and the percentage on the corresponding total number is given in bracket. (**c**) The co-mutational groups between the MAGO and Y14 families. The 'Conserved' indicates that the functional domains sites in the groups were not changed in both proteins. The 'Changed' indicates that the functional domains sites in the groups were changed in one of the two proteins. The number of the co-mutational groups is given out of the bracket and the percentage on the total number is given in the bracket. M and Y include the co-changed residues in the functional domains of both MAGO (M) and Y14 (Y) families, while M or Y is groups that the residues from one protein family were changed. The groups in red were formed by the clade-specific residues (Table S5; Figure 6).

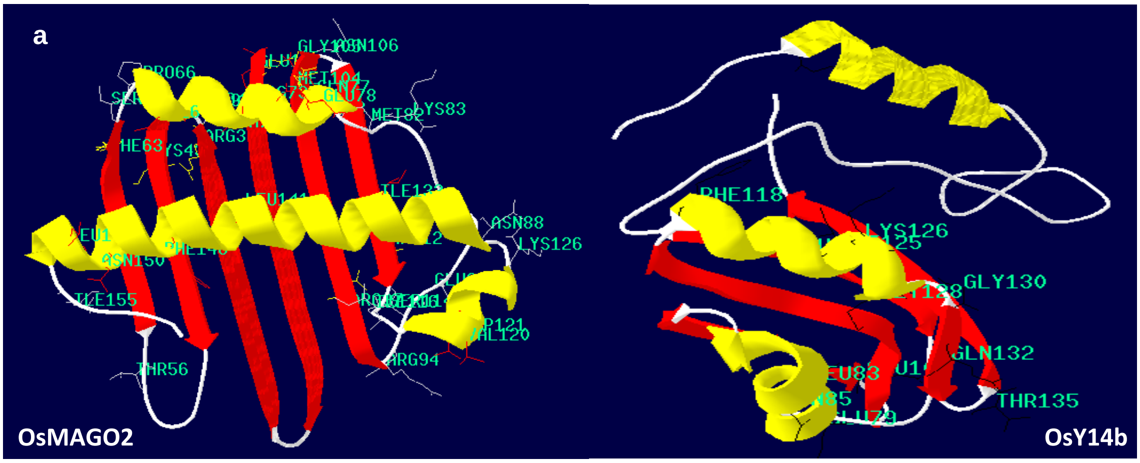

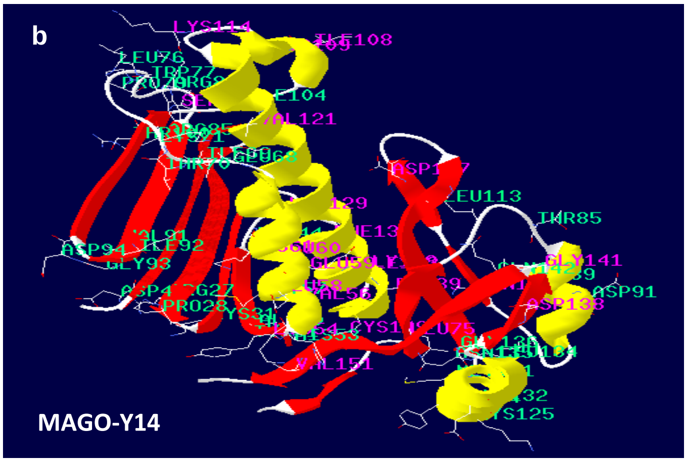

c

	MAGO-CORE (36)	Y14-RBD (12)
helix	12 (33.3%)	6 (50%)
sheet	11 (30.6%)	3 (25%)
Random coil	13 (36.1%)	3 (25%)

Figure S4. Location of the correlated residues on the tertiary structures of the MAGO and Y14 proteins. (**a**) Distribution of the correlated residues on the rice proteins. OsMAGO2 and OsY14b were set as templates. The co-evolved sites predicted by CAPS or the clade-specific sites were highlighted as amino acid (AA) followed by its position. (**b**) Location of the correlated residues on the MAGO-Y14 structure. The crystallized structure of human Magoh-Y14 was set as a template [30]. The residues distributed in the interaction interface of the MAGO-Y14 complex are highlighted in meganta. 17 (P_{66}, V_{68}, L_{69}, A_{72}, R_{73}, Q_{77}, E_{78}, V_{120}, D_{121}, Y_{126}, I_{133}, L_{141}, F_{146}, N_{150}, L_{151} and I_{155}) of the 36 co-evolved sites in the MAGO-core corresponding to OsMAGO2, and 7 (P_{66}, R_{70}, A_{72}, I_{116}, D_{121}, I_{133} and N_{150}) of 15 clade-specific sites were in the interacting interface. Corresponding to OsY14b, 4 (L_{69}, E_{101}, Q_{132} and T_{135}) of 12 coevolved sites in Y14-RBD, and 5 (D_{95}, A_{107}, R_{136}, A_{143} and S_{145}) of 12 clade-specific sites were also in the interacting interface. (**c**) The distribution of the co-evolved residues in the interaction interface and the minimal interaction domains. The interacting interfaces were helixes (in yellow) and random coils (in white) in the MAGO-core ($AA_{66\text{-}78}$, and $AA_{114\text{-}158}$) and sheets (in red) and random coils in the Y14-RBD ($AA_{58\text{-}73}$, $AA_{95\text{-}115}$ and $AA_{132\text{-}149}$), respectively. They largely overlapped with the minimal interaction domains that we determined in yeast. Thus, the co-evolution between the MAGO and Y14 families is required for the maintenance of their obligate heterodimerization mode.

Table S1. Primer information for protein-protein interactions in yeast two-hybrid assays.

Gene name	Sequences (5'-3')	
OsMAGO1	F:ATGGCGACGGTGGCCGGC;	R:TGAAAGGAGCTCAAGATTGA;
OsMAGO2	F:ATGGCGACGGGCGGCGCC;	R:TGAAAGGAGCTCAAGATTGA;
OsMAGO1	F:ATCCATGGCGACGGTGGCCGGC;	R: TGGATCCTGAAAGGAGCTCAAGATTGA;
OsMAGO2	F:ATCCATGGCGACGGGCGGCGCC;	R: ATGGATCCTGAAAGGAGCTCAAGATTGA;
OsY14a	F:ATCCATGGCTGCGGTGACCAACGC;	R: ATGGATCCTCAGTATCTTCTCCTCGGTG;
OsY14b	F:AACCATGGCGGCGGCGGCGGAGGACG;	R: ATGGATCCAGTCAACATGTCAAGGCAG;
AtMAGO	F:ATCCATGGCCGCGGAAGAAGCGACG;	R:ATGGATCCTAGATAGGCTTGATTTTGAAG;
AtY14	F:ATCCATGGTCAGTCGGCGAAACGGGAAG;	R:ACGGATCCCAAATCATACAATCAGTAACG;
SlMAGO	F:ATCCATGGACAGAAGAGGGCAAACTATG;	R:AAGGATCCAGCTAACGTTTAACATACGC;
SlY14	F:AACCATGGCGAACCCGGAGGAGGCTG;	R:ATGGATCCTTGCAAATAGGTGTGTGC;
Pp/PfMAGO1	F:GGCCATGGGGGAATTGGAAGAGAATG;	R:ATGGATCCGGGTTTGATCTTGAAATGGAG;
Pp/PfMAGO2	F:GGCCATGGGGGAGATGGCAGAGAAC;	R:ATGGATCCGGGTTTGATCTTGAAATGGAG;
Pp/PfY14	F:ATCCATGGGGAACGGGGAGGAGGCAG;	R:ATGGATCCTTAGTATCTCCTCCTGGGACTTC;
CeMAGO	F:ATCCATGGACATGAGCGGCGAAGAAGAGAAG;	R:ATGGATCCGATTGGCTTGATCTTGAAGTG;
CeY14	F:ATCCATGGACATGAGCGACAACGAAGTAGAG;	R:ATGGATCCTCAGCGCTTTCCAGAAGTCTTC;
DmMAGO	F:ATCCATGGTCATGTCCACGGAGGACTTTTAC;	R:ATGGATCCGGCTACGGCTTATATGGGCTTG;
DmY14	F:ATCCATGGCCGATGTGTTGGACATTG;	R:ATGGATCCTTATCTGCGACGCTTTTCGGAC;
HsMAGO	F:ATCCATGGCTGTGGCTAGCGATTTC;	R:CCGAATTCACAAACAGCCTGAAAACATAC;
HsY14	F:ATGAATTCATGGCGGACGTGCTAGATCTTC;	R:ATGGATCCTCAGCGACGTCTCCGGTCTGG;
MmMAGO	F:ATCCATGGTATAGCGGGCTTTACCGAGG;	R:CCGAATTCAAAACATCCCATTCAAATTGG;
MmY14	F:CCGAATTCAGCGCCGAGAGAAGCAAGATG;	R:ACGGATCCGAGACCACCCAGTCGACAGAG;
OsLFY	F:ATCATATGGATCCCAACGATGCCTTCTCG;	R:ATGAATTCTCCGGCGAGCTTAGAACAAGGG;
AtLFY	F:ATCCATGGATCCTGAAGGTTTCACGAG;	R:ATGAATTCTAGAAACGCAAGTCGTCGCC;
AtMAGOm64	F: CTCAAGGAAGCCAAGCGTATCGTCTCCGAG;	R: CTCGGAGACGATACGCTTGGCTTCCTTGAG;
AtMAGOm142	F:ATCCATGGCCGCGGAAGAAGCGACG;	R: AGGCTTGATTTTGAAGTGCAGATTGATGAG;
AtY14m154	F: AGCGCTATTAAAGCAATGAATGGTGCTGAG;	R: CTCAGCACCATTCATTGCTTTAATAGCGCT;

Table S2. Primer information for determining the minimal interaction domains of the rice MAGO and Y14.

Usage	Primer name	Sequence 5'-3'
OsMAGO1 deletion assay	*OsMAGO1delF1*	F:ATCCATGGACGATGGCGGCGGCGGCGGT;
	OsMAGO1delF2	F:ATCCATGGAGTTCTACCTGCGGTACTAC;
	OsMAGO1delF3	F:ATCCATGGTGGGGCACAAGGGGAAGTTC;
	OsMAGO1delF4	F:ATCCATGGGGCACGAGTTCCTGGAGTTC;
	OsMAGO1delF5	F:ATCCATGGAGTTCGAGTTCCGGCCCGAC;
	OsMAGO1delF6	F:ATCCATGGGGAAGCTCCGGTACGCCAAC;
	OsMAGO1delF7	F:ATCCATGGCCAACAACTCCAACTACAAGAAG;
	OsMAGO1delF8	F:ATCCATGGACACCATGATCCGCAAGGAG;
	OsMAGO1delF9	F:ATCCATGGTGTTCGTCTCCCCCTCC;
	OsMAGO1delF10	F:ATCCATGGTCCTCCGCGAGGCCACCAGG;
	OsMAGO1delR1	F:ATGGATCCTCAAATGGGCTTGATCTTGAAGTG;
	OsMAGO1delR2	F:ATGGATCCTCAGAGATTGATGAGTGAAAACAC;
	OsMAGO1delR3	F:ATGGATCCTCAGAAGCACTTAAGATCCTGGAC;
	OsMAGO1delR4	F:ATGGATCCTCAGAGGTAGTAGAAGATGCGGAG;
	OsMAGO1delR5	F:ATGGATCCTCAGCCCTCGGGGTCCTGGCTGGT;
	OsMAGO1delR6	F:ATGGATCCTCACTGGACGTCGACGAGGGAGCC;
	OsMAGO1delR7	F:ATGGATCCTCAGATCTTGGAGGTGGTGAAGGA;
	OsMAGO1delR8	F:ATGGATCCTCAGATGTGCTCGTTCCCCATCAC;
	OsMAGO1delR9	F:ATGGATCCTCAGATCTCCAGCTCCTGCCTTCC;
	OsMAGO1delR10	F:ATGGATCCTCACACGCGGTCTGGCTCCGGCCA;
OsMAGO2 deletion assay	*OsMAGO2delF2*	F:ATCCATGGAGTTCTACCTGCGGTACTAC;
	OsMAGO2delR2	F:ATGGATCCTCAGAGATTGATGAGTGAAAACAC;
OsY14a deletion assay	*OsY14adelF1*	F:ATCCATGGTGCTAGTCACTGGGGTTCAT;
	OsY14adelF2	F:ATCCATGGAAGAAGCTCAGGAAGATG;
	OsY14adelF3	F:ATCCATGGATCTTCACAATATTTTCCGG;
	OsY14adelF4	F:ATCCATGGACTTTGGGCAGGTCAAGAAC;
	OsY14adelF5	F:ATCCATGGATCGCCGAACTGGATTCGTG;
	OsY14adelF6	F:ATCCATGGTGAAGGGATATGCTCTTATTG;
	OsY14adelF7	F:ATCCATGGAGTATGAAACTTTCGAGG;
	OsY14adelF8	F:ATCCATGGCTCAGGCTGCAATAAAAGC;
	OsY14adelF9	F:ATCCATGGCATTGGATGGAACTGAG;
	OsY14adelF10	F:ATCCATGGAGCTTCTAACACAAATC;
	OsY14adelR1	F:ATGGATCCTCAGGATCTGGAGCGTCTTGGTG;
	OsY14adelR2	F:ATGGATCCTCATCTCTTCCGAATATTCCTGCG;
	OsY14adelR3	F:ATGGATCCTCACTTGACAGGGCCATTACTAAATGC;
	OsY14adelR4	F:ATGGATCCTCACCA ATCAACACTTATGATTTG;
	OsY14adelR5	F:ATGGATCCTCATGTTAGAAGCTCAGTTCCATC;
	OsY14adelR6	F:ATGGATCCTCACAATGCTTTTATTGCAGCCTG;
	OsY14adelR7	F:ATGGATCCTCAAGCTTCCTCGAAAGTTTCATAC;
	OsY14adelR8	F:ATGGATCCTCACTCAATAAGAGCATATCCC;
	OsY14adelR9	F:ATGGATCCTCACTTCACGAATCCAGTTCGGCG;
	OsY14adelR10	F:ATGGATCCTCAATCCAAATTCAAATGCAAG;
OsY14b deletion assay	*OsY14bdelF1*	F:ATCCATGGGCCCACAGCGCTCTATTGAAG;
	OsY14bdelF2	F:ATCCATGGGATGGATACTACTGGTCTCTG;
	OsY14bdelF3	F:ATCCATGGGAGTCAAAGAAGACGCGGAAG;
	OsY14bdelF4	F:ATCCATGGAAGATGATCTGTACAACAC;
	OsY14bdelR1	F:ATGGATCCTCATAATGGCCTTGTACTGGTGAG;
	OsY14bdelR2	F:ATGGATCCTCAGAGTTTCTGTATAGGACCTCTG;
	OsY14bdelR3	F:ATGGATCCTCAGCTGAATGCCCAGTCAACATAG;
	OsY14bdelR4	F:ATGGATCCTCAGACAGTCCTTGTTAACAGCTG;

Table S3. Information on sequences used in the present work.

Species	*MAGO*		Gene structure		*Y14*		Gene structure		*LFY*	
	Gene name	Accession number	Exon number	Intron number	Gene name	Accession number	Exon number	Intron number	Gene name	Accession number
Arabidopsis thaliana	*Ath*	KF051000	3	2	*Ath*	KF051012	3	2	*Ath*	KF051022
Populus trichocarpa	*Ptr*	EF144848	3	2	*Ptr-a*	XM_002299753	4	3	*Ptr*	XM_002322271
			3	2	*Ptr-b*	XM_002314049	4	3		
Physalis floridana	*Pfl-1*	KF051010	3	2	*Pfl*	KF051015	nd	nd	*Pfl*	Unigene87481P106
	Pfl-2	KF051007	3	2						
P.philadelphica	*Pph-1*	KF051009	nd	nd	*Pph*	KF051014	nd	nd	*Pph*	nd
	Pph-2	KF051006	nd	nd						
Solanum lycopersicum	*Sly -1*	KF051001	3	2	*Sly*	KF051013	4	3	*Sly*	Solyc03g118160
Gossypium raimondii	*Sly-2*	Gorai.007G085400	3	2	*Gra-a1*	Gorai.013G253000	4	3	*Gra*	Gorai.001G053900
	Gra	Gorai.004G069800	3	2	*Gra-a2*	Gorai.006G125200	4	3		
					Gra-b	Gorai.009G077300	4	3		
Medicago truncatula	*Mtr-1*	Medtr3g163680	3	2	*Mtr*	Medtr5g008930	4	3	*Mtr*	XM_003602697
	Mtr-2	Medtr3g132960	3	2			4	3		
Cucumis sativus	*Csa*	Cucsa.259980	3	2	*Csa*	Cucsa.055310	4	3	*Csa*	XM_004137968
Prunus persica	*Ppe*	ppa012866m	3	2	*Ppe*	ppa007553m	4	3	*Ppe*	EF175869
Mimulus guttatus	*Mgu*	mgv1a015616m	3	2	*Mgu-a*	mgv1a014186m	4	3	*Mgu*	AY524036
			3	2	*Mgu-b*	mgv1a01307m	4	3		
Vitis vinifera	*Vvi*	XM_002281258	3	2	*Vvi*	XM_002281192	4	3	*Vvi*	XM_002284628
Zea mays	*Zma-1*	NM_001152441	3	2	*Zma-a1*	NM_001157087	4	3	*Zma-1*	DQ343237
	Zma-2	NM_001153494	3	2	*Zma-a2*	NM_001156826	4	3	*Zma-2*	AY789045
					Zma-b	NM_001156791	4	3		
Sorghum bicolor	*Sbi-1*	XM_002443679	3	2	*Sbi-a1*	XM_002439204	4	3	*Sbi*	XM_002446991
	Sbi-2	XM_002462494	3	2	*Sbi-a2*	XM_002466188	4	3		
					Sbi-b	XM_002457800	4	3		
Setaria italica	*Sit-1*	Si014566m	3	2	*Sit-a*	Si023278m	4	3	*Sit*	Si011756m
	Sit-2	Si032825m	2	1	*Sit-b*	Si037569m	4	3		
Brachypodium distachyon	*Bdi-1*	XM_003573221	3	2	*Bdi-a1*	XM_003568923	4	3	*Bdi*	XM_003580387
	Bdi-2	XM_003573221	3	2	*Bdi-a2*	XM_003559220	4	3		
					Bdi-b	Bradi2g133m	4	3		
Oryza_sativa	*Osa-1*	KF051011	3	2	*Osa-a*	KF051016	4	3	*Osa*	KF051023
	Osa-2	KF051008	3	2	*Osa-b*	KF051017	4	3		
Hordeum vulgare	*Hvu*	AK252690	nd	nd	*Hvu-a*	AK250786	nd	nd	*Hvu*	MLOC_14305
					Hvu-b	MLOC_64220	nd	nd		
Phoenix dactylifera	*Pda*	30s1136351g005	3	2	*Pda-a*	30S975321g003	4	3	*Pda-1*	30s795141g001
					Pda-b	30s701331g002	4	3	*Pda-2*	30s854671g005
Musa acuminata	*Mac*	Achr11T21790	3	2	*Mac*	Achr6G08340	5	4	*Mac*	Achr6T16390
Picea sitchensis	*Psi*	EF082784	nd	nd	*Psi*	EF086042	nd	nd	*Psi*	EF087799
Marsilea vestita	*Mve*	AF329672	nd	nd	*Mve*	EU009956	nd	nd	*nd*	nd
Physcomitrella patens	*Ppa-1*	XM_001770356	3	2	*Ppa-a*	XM_001758204	3	2	*Ppa-1*	XM_001762002
	Ppa-2	XM_001763749	3	2	*Ppa-b*	XM_001771246	3	2	*Ppa-2*	XM_001765249
Chlamydomonas reinhardtii	*Cre*	XM_001694693	4	3	*Cre*	XM_001696940	2	1	*nd*	nd
Volvox carteri	*Vca*	Vocar20010354m	4	3	*Vca*	XM_002953371	2	1	*nd*	nd
Caenorhabditis elegans	*Cel*	KF051002	2	1	*Cel*	KF051018	3	2	*nd*	nd
Drosophila melanogaster	*Dme*	KF051003	2	1	*Dme*	KF051019	2	1	*nd*	nd
Danio rerio	*Dre*	NM_001017700	5	4	*Dre*	NM_001013345	6	5	*nd*	nd
Xenopus tropicalis	*Xtr*	XM_002931425	5	4	*Xtr*	NM_001045682	4	3	*nd*	nd
Taeniopygia guttata	*Tgu*	NM_001245420	5	4	*Tgu*	NM_001245281	5	4	*nd*	nd
Mus musculus	*Mmu*	KF051005	5	4	*Mmu*	KF051021	6	5	*nd*	nd
Homo sapiens	*Hsa*	KF051004	5	4	*Hsa*	KF051020	6	5	*nd*	nd
*Pan troglodytes **	*Pt*	XM_520743	5	4	*Pt*	XM_003806189	6	5	*nd*	*nd*
*Aedes aegypti**	*Aae*	XM_001660782	2	1	*Aae*	XM_001652117	1	0	*nd*	*nd*
*Apis mellifera**	*Ame*	XM_001120074	1	0	*Ame*	XM_395245	4	3	*nd*	*nd*
*Wuchereria bancrofti**	*Wba*	ADBV01001636.1	3	2	*Wba*	ADBV01000095.1	2	1	*nd*	*nd*
*Loa loa**	*Llo*	XP_003144197	3	2	*Llo*	EJD75995	nd	nd	*nd*	*nd*

nd: no data
* indicate the sequences in the species were not used to construct the tree

Table S4. Quality factors of different protein modeling.

	**OsMAGO1**	**OsMAGO2**	**OsY14a**	**OsY14b**
Modeled residue range	14-156	16-158	23-113	12-146
Based on template (Parent PDB)	1oo0 Chain A X-Ray, 1.85 Å	1oo0 Chain A X-Ray, 1.85 Å	p27 Chain: B X-Ray, 2.00Å	1hl6 Chain: A X-Ray, 2.50Å
Sequence identity (%)	83.92	84.62	63.74	35.56
E-value	2.60e-65	1.83e-66	2.39e-33	6.30e-24
QMEANscore4 (0-1)	0.738	0.772	0.863	0.657
Quality information (QMEAN Z-Score)	-0.64	-0.26	0.35	-1.51
Template	*Drosophila* MAGO NASHI protein [15]	*Drosophila* MAGO NASHI protein [15]	Human Y14 protein [30]	*Drosophila* Y14 Protein [32]

Table S5. The correlated mutation residues between the MAGO and Y14 families.

Group	MGAO		Y14			Mean Dc ±SE	Mean ρ±SE
	Nt	MAGO-Core (16-158)	Nt	Y14-RBD (69-139)	Ct		
G1	A6		V8		P166	4.39±1.55	0.40±0.03
G2	G13		V8, D27, R44, H 58	K126	P166	5.37±0.92	0.36±0.01
G3		S114	D27			5.46±2.45	0.39±0.00
G4		P66		N85	A143	6.19±2.70	0.40±0.05
G5		N150		K 126		3.41±0.78	0.44±0.00
G6		K83		I 125		5.02±2.42	0.54±0.00
G7	A2		E17, D 27	L83		5.01±1.48	0.44±0.02
G8		R94	L33	L83	L152	6.11±2.51	0.51±0.11
G9		G105, L69		G130		3.72±1.62	0.44±0.05
G10		R39	A2, D27	Q132		4.87±0.77	0.43±0.04
G11		K43	D27, S49	M128, G130		6.12±2.36	0.41±0.04
G12		M82	E9, D27, G57	L69, E79, N85, F118, T135	A143, S145, T153, S162, L169	7.91±0.85	0.53±0.04
G13	A7		V8, D27			2.17±0.59	0.35±0.02
G14		R97	E17, D22, S49	E101		5.07±1.09	0.66±0.07
G15		L151	D27, L33		T155	5.99±2.72	0.51±0.09
G16		R73	D16, D27, D28, R31			4.77±1.04	0.38±0.03
G17		I133, E16, T112	A2	Q132		5.11±0.93	0.46±0.03
G18		E91	E9, A23, D27, G57, G59	E79, N85, I125, T135	S145, T153, S162, R163, R167, R168, 169L, A170	7.26±0.57	0.46±0.02
G19		F146	A2			4.26±0.73	0.57±0 .00
G20		V103	S49	G130		4.15±0.86	0.47±0.09
G21		E78		L83		5.21±3.54	0.53±0.00
G22	G15		R15			9.47±3.55	0.41±0 .00
G23		V120,Y126		Q132		5.69±2.87	0.36±0.01
G24		S65	A3, A5, E6, G57	T135	A143, G147, K151	3.16±1.09	0.52±0.04
G25		L141	R35			4.60±0.75	0.42±0 .00
G26		P40	D27		P157	8.49±1.92	0.45±0.09
G27		T56	A23, D27, G57, G59	E79, N85	S153, T155, P157, S162, R163, R167, L169, A170	7.61±0.66	0.42±0.02
G28		V68	E17	L83, E101, F118		5.14±2.32	0.38±0.01
G29		I155	L33			7.38±4.77	0.34±0 .00
G30		M104	R62			6.21±0.78	0.39±0 .00
G31		F63	D28, S49	E79, M128, G130	A143	5.28±0.87	0.40±0.04
G32	F11		D27, R44			4.94±1.11	0.43±0.07
G33		N106	D27	E79, N85	S145, T153, T155, P157, S162, L169	7.84±0.66	0.44±0.03
G34	T3		D27		L169	4.79±1.38	0.36±0.00
G35		N88	E9, A23, D27, N55	L69, E79, N85, F118, T125, T135	A143, S145T153, S162, R163, L169, A170	7.65±0.79	0.51±0.03
G36	D10		D27			2.11±0.90	0.37±0 .00
G37		Q77	V8, D27, R44, H58	K126		5.62±1.64	0.38±0.02
G38	G4,P12		D27, R40			3.89±0.97	0.41±0.04
G39		D121	D27, R62		T155	8.42±1.81	0.49±0.08
G40		I116		I125	T155	11.69±3.43	0.68±0.26
G41		A72	R15, D27, N55			11.45±1.88	0.53±0.13

Nt: N-terminal; Ct: C-terminal; ρ: Pearson correlation coefficient; Dc: amino acid site variance; SE: standard error; RBD: RNA binding domain; The amino acids and positions were defined with OsY14b and OsMAGO2 as references, respectively. The group marked in green indicate the group that their residues were co-altered in both functional domains of Y14 and MAGO. The sites marked in red mean they were strictly clade-specific, the blue mean the amino acids changed but were not clade specific.
